# GPX4 knockdown suppresses M2 macrophage polarization in gastric cancer by modulating kynurenine metabolism

**DOI:** 10.7150/thno.108817

**Published:** 2025-04-22

**Authors:** Jingli Xu, Chunyan Weng, Yanqiang Zhang, Qianyu Zhao, Jiahui Chen, Siwei Pan, Yan Wang, Ruolan Zhang, Yuqi Wang, Weiwei Zhu, Mengxuan Cao, Dan Zu, Shengjie Zhang, Zhiyuan Xu, Can Hu, Xiangdong Cheng

**Affiliations:** 1 Zhejiang Chinese Medical University, Hangzhou, Zhejiang 310053, China; 2 Department of Gastric Surgery, Zhejiang Cancer Hospital, Hangzhou Institute of Medicine (HIM), Chinese Academy of Sciences, Hangzhou, Zhejiang 310022, China; 3 Zhejiang Provincial Research Center for Upper Gastrointestinal Tract Cancer, Zhejiang Cancer Hospital, Hangzhou 310022, China.; 4 Hangzhou Institute of Medicine (HIM), Chinese Academy of Sciences, Hangzhou, Zhejiang 310022, China

**Keywords:** GPX4, gastric cancer, TAM polarization, kynurenine metabolism, KYNU, ubiquitin

## Abstract

**Background:** Glutathione peroxidase 4 (GPX4), an important factor regulating redox homeostasis, plays an important role in tumor microenvironment and progression. However, the role of GPX4 in gastric cancer (GC) is unclear.

**Methods:** Spectral flow cytometry and multiplex immunohistochemistry were employed to assess the correlation between GPX4 expression and immune cell infiltration. Metabolomics analysis of conditioned media from GPX4 knockdown NUGC3 cells identified metabolic alterations. Additionally, both in vitro and in vivo functional studies were conducted to elucidate the mechanistic role of GPX4 in regulating the tumor microenvironment and progression.

**Results:** Knockdown of GPX4 in GC cells inhibited tumor growth, enhanced CD8^+^ T cell infiltration, and suppressed the polarization of tumor-associated macrophages (TAMs) toward the pro-tumor M2 phenotype. Multiplex immunohistochemistry revealed a positive correlation between GPX4 expression and M2 macrophage infiltration in clinical samples from patients with GC. Metabolomics revealed that GPX4 knockdown regulate kynurenine metabolism pathway. Furthermore, mechanistic studies reveal that GPX4 silencing elevates lipid peroxidation, triggering the conversion of KYNU ubiquitin chain modifications from K48 to K63. Such ubiquitination remodeling stabilizes KYNU expression (a key kynurenine-metabolizing enzyme), reduces kynurenine accumulation, and ultimately reprograms TAM polarization to enhance antitumor immunity. We also identified that the K96 and K163 sites are important for KYNU's modification by K48 and K63 ubiquitin chains.

**Conclusion:** Our study not only affirm the role of GPx4 in GC progression but also highlight it as a promising target for reshaping the immune microenvironment.

## Introduction

Gastric cancer (GC) represents the fifth and fourth malignancy globally in terms of incidence and mortality, respectively, with particularly high incidence rates in East Asia 1,2. GC diagnosis often occurs at an advanced stage, with cases losing the opportunity for surgical intervention and facing poor prognosis. In addition to surgery, chemotherapy remains the primary therapeutic option for advanced GC, supplemented by immunotherapy, targeted therapy, and other comprehensive treatment strategies 3. However, non-surgical treatment options for advanced gastric cancer are often limited due to tumor heterogeneity and complexity 4. Some patients exhibit chemotherapy resistance or severe chemotherapy-related complications. Furthermore, only 10-20% of advanced gastric cancer patients benefit from immunotherapy. Moreover, few targeted therapeutic options are available for GC, with trastuzumab being the only first-line therapy for HER2-positive cases, who constitute 20% or less of the total patient population 5. Thus, developing new targeted therapies or combining existing treatments to improve patient prognosis in gastric cancer remains a critical focus in the management of this disease.

Glutathione peroxidase 4 (GPX4), a selenocysteine-containing protein, catalyzes the reduction of phospholipid hydroperoxides (PLOOH) to corresponding alcohols, exerting potent antioxidant activity and playing a central regulatory role in ferroptosis 6. GPX4 expression is significantly elevated in various tumors, including breast cancer 7, colorectal cancer 8, lung cancer 9, and liver cancer 10. Overexpression of GPX4 in tumor cells inhibits ferroptosis by neutralizing lipid peroxides, promoting tumor survival and metastasis 11. Moreover, recent studies have demonstrated that GPX4 regulates the metastasis of GC cells by modulating RCC2 expression 12. Conversely, targeting GPX4 or inhibiting it with small molecules like RSL3 induces ferroptosis in cancer cells, effectively overcoming tumor resistance 13. Our previous research showed a significant increase in GPX4 in GC, and the use of small-molecule inhibitors targeting GPX4 significantly suppressed GC progression 14. Interestingly, previous studies have shown that GPX4 expression in cancer and immune cells in the tumor microenvironment (TME) is not uniform, but rather exhibits different immunosuppressive or immunoenhancing functions depending on the context 15,16. Previous studies showed that inhibiting GPX4 results in T cell activation in mouse models of colorectal cancer; However, tumor cells subsequently overexpress PD-L1 and there was an increase in myeloid-derived suppressor cells (MDSCs) in the immune microenvironment, which impaired the cytotoxic activity of these T cells against tumor cells 17. Conversely, other research suggests that elevated GPX4 expression in colorectal cancer enhances the infiltration of CD4^+^ and CD8^+^ T cells and improves the efficacy of PD-1 inhibitors. This may be attributed to GPX4's modifications of N6-methyladenosine (m6A) and 5-methylcytosine (m5C), which activate STING and help maintain redox balance during cancer immunotherapy, thereby facilitating immune regulation 18. In previous studies, we found that GPx4 was highly expressed in GC and was associated with GC progression and metastasis 14,19. However, the effect of GPX4 expression on the immune microenvironment of gastric cancer was still unclear.

Within the tumor microenvironment (TME), malignant cells dynamically interact with stromal components, extracellular matrix proteins, and metabolic byproducts to drive tumor progression, metastasis, and therapeutic resistance 20. These interactions sustain proliferative signaling, promote angiogenesis, and facilitate immune evasion through multifaceted mechanisms. For instance, hypoxia-induced HIF-1α activation transcriptionally upregulates the secreted protein ESM1, which enhances the Warburg effect and promotes vasculogenic mimicry in ovarian cancer by stabilizing PKM2 SUMOylation and STAT3 phosphorylation 21. Similarly, MYC-driven transcriptional programs in colorectal cancer upregulate tryptophan transporters (SLC7A5/SLC1A5) and metabolic enzymes (AFMID), amplifying kynurenine pathway activity to fuel tumor growth—a process suppressible by pharmacological inhibition of kynurenine-AHR axis interactions 22. Emerging therapeutic strategies targeting TME components, such as cancer-associated fibroblasts (CAFs) and tumor-associated macrophages (TAMs), demonstrate promising antitumor efficacy 20. TAMs are a crucial part of the TME, impacting tumor growth, angiogenesis, immune regulation, metastasis, and drug resistance 23. Upon activation, macrophages may be grouped into the M1 (expressing CD80/86) and M2 (expressing CD206, CD163, CD204) phenotypes 24. Generally, M1 macrophages induce inflammatory responses that target invading pathogenic organisms and cancer cells via secretion of pro-inflammatory cytokines and chemokines, including IL-12, TNF-α, CXCL-10, and IFN-γ 25,26. In contrast, M2 macrophages produce various anti-inflammatory cytokines, e.g., IL-10, IL-13, and IL-4, exerting immunosuppressive effects that favor tissue repair and tumor progression 25,26. The heterogeneity of the TME across different regions results in distinct inflammatory signals and cytokines, which are the main drivers of macrophage polarization into different subtypes 27. Notably, the uneven distribution of tumor metabolism is a significant influencing factor. Macrophages surrounding perfused blood vessels, where high levels of glucose, glutamine, and oxygen are present, are polarized towards the M1 phenotype; conversely, those in regions of chronic hypoxia and high lactate concentrations, far from blood vessels, are induced to polarize towards the M2 phenotype 28,29. Therefore, tumor metabolites play an important role in regulating the immune microenvironment.

In this study, we utilized metabolomics to reveal that knocking down GPX4 in GC cells reduces M2 macrophage polarization within the TME by regulating kynurenine levels, thereby inhibiting gastric cancer growth. Further results demonstrated that GPX4 knockdown in GC cells induces a shift in the ubiquitination of the key kynurenine metabolism enzyme KYNU, thereby stabilizing KYNU protein levels.

## Methods

### Transcriptome sequencing and differential expression analysis

Transcriptome sequencing was performed on 60 pairs of GC and adjacent noncancerous tissue samples from Zhejiang Cancer Hospital. RNA extraction and library preparation were conducted, followed by sequencing on the Illumina NovaSeq 6000 platform. Sequencing data processing was followed by alignment to the human genome. Differential expression analysis of ferroptosis-related genes utilized the DESeq2 package. Significant genes were identified with adjusted P < 0.05.

### Multiplex immunohistochemistry and immunohistochemistry

Primary GC tissue samples from 179 cases administered gastrectomy were obtained at Zhejiang Cancer Hospital between 2007 and 2017. TMAs were constructed for further analysis. IHC was conducted with anti-GPX4 primary antibodies (Abcam, cat. No. ab125066) by standard protocols. GPX4 expression score was evaluated as the product of staining intensity (0 to 3 representing no, weak, intermediate and strong signals, respectively) and percent positive cancer cells (0 to 4 representing < 5%, 5-25%, 26-50%, 51-75% and 76-100%, respectively), with total scores (H scores) of 0 to 12. Multiplex immunohistochemistry (mIHC) was performed with specific primary antibodies to access the expression of CD68 (Abcam, cat. No. ab955), CD86 (CST, cat. No. 91882S), CD206 (Abcam, cat. No. ab252921) in TMAs. Multiplex staining was achieved by sequentially stripping and re-probing the tissues with different primary antibodies. Counterstaining was conducted with hematoxylin, followed by dehydration and mounting. The sections were then scanned and analyzed with a digital pathology platform to quantify marker expression levels. Based on GPX4's median H-score and median amounts of M1 and M2 macrophages, the specimens were grouped into the high- and low-expression groups. Two experienced pathologists scored the samples independently in a blinded fashion. Ethical approval was obtained from Zhejiang Cancer Hospital (approval number: IRB-2023-960(IIT)).

### Cell culture and animal housing

Human GC (NUGC3 and NUGC4), mouse GC (MFC) and human monocytic (THP-1) cells were obtained from Chinese Academy of Sciences (Beijing, China) and maintained in RPMI 1640 containing 10% fetal bovine serum (FBS) at 37 °C in a humid environment containing 5% CO₂. Cell passaging was conducted every 2 days. The animal study had approval from the Institutional Animal Care and Use Committee (IACUC) of Zhejiang Chinese Medical University. Six-week-old male 615 were housed under specific pathogen-free (SPF) conditions using a 12-h photoperiod, with ad libitum standard chow and water. Ethical approval was obtained from Zhejiang Chinese Medical University (approval number: IACUC-20211025-11).

### Western blot and quantitative real-time PCR (RT-qPCR) analysis

Western blotting was carried out to evaluate protein levels. Cell lysis was conducted with RIPA buffer containing protease inhibitors. Equal amounts of protein underwent separation by SDS-PAGE, followed by electro-transfer onto a PVDF membrane. Upon blocking, successive incubations were conducted with primary antibodies targeting GPX4 (Abcam, cat. No. ab125066), Vinculin (Abcam, cat. No. ab129002), IDO1 (Abcam, cat. No. ab211017), IDO2 (Abcam, cat. No. ab307144), H3 (Proteintech, cat. No. 68345-1-Ig), KYNU (Proteintech, cat. No. 11796-1-AP), AhR (Proteintech, cat. No. 67785-1-Ig), STAT3 (Abcam, cat. No. ab68153) and p-STAT3 (Abcam, cat. No. ab267373) and HRP-linked secondary antibodies. The ECL detection system was employed for visualization, and data analysis utilized the ImageJ software.

qRT-PCR was conducted for gene expression assessments. Total RNA extraction was carried out, and cDNA was prepared. qRT-PCR was performed with the SYBR Green qPCR Master Mix kit as directed by the manufacturer (ES science, cat no. QP002). Specific primers for IDO1, KYNU, IL-10, TGF-β, TNF-α, VEGFA, MMP9, and GAPDH (and) were listed in Table 1, and the 2^-∆∆Ct^ method was employed for data analysis.

### Lentiviral infection and plasmid transfection

Lentiviral infection and plasmid transfection were used to knockdown or overexpress target genes. All the lentiviruses and plasmids employed in this study were constructed by GeneChem (Shanghai, China). Lentiviral infection and plasmid transfection were conducted according to the manufacturer's instructions. For GPX4 knockdown/knockout and overexpression, lentiviral vectors carrying shRNA/sgRNA sequences and overexpression constructs, respectively, were used to infect NUGC3 or MFC cells, followed by puromycin selection to establish stable cell lines. For GPX4 knockdown assays, human-GPX4 shRNA-1# and shRNA-2# target sequences were GTGGATGAAGATCCAACCCAA and GTGAGGCAAGACCGAAGTAAA, mouse-GPX4 shRNA-1# and shRNA-2# target sequences were ATGCCATCAAATGGAACTTTA and ACAGCAAGATCTGTGTAAATG. For GPX4 knockout assays, human-GPX4 sgRNA target sequence was AGCCCCGCCGCGATGAGCCT; Following these procedures, cells were selected for monoclonal isolation and were named KO-1# and KO-2#. For KYNU knockdown, siRNAs target sequences were GAUAAGCUGAGGCACUUCA and AAUACAGGAUCUGCCUCCA. SiRNAs underwent transfection into NUGC3 cells with Lipofectamine™ 3000 (Invitrogen, cat. No. L3000075). For IDO1 overexpression, plasmids encoding the full-length IDO1 gene were transfected into NUGC3 cells. Silencing and overexpression efficiencies were assessed by RT-qPCR and immunoblot.

### Tumor xenograft model and spectral flow cytometry analysis

MFC cells (1×10^5^) were resuspended in 100 µL of PBS and injected by the subcutaneous route into the mouse's right flank. After 3 weeks, with tumor volume approximating 500-1000 mm³, tumors were excised and cut into small fragments of 1-2 mm³. These fragments were then adhered to the stomach's greater curvature in mice using biological glue. During this period, mouse body weight measurements occurred twice weekly, and the animal's general condition and behavior were closely monitored. Two weeks later, euthanasia was carried out, and tumors were photographed and weighed. Tumor tissues underwent dissociation using tissue dissociation reagents (Miltenyi Biotec, cat. No. 130-096-730) as directed by the manufacturer. Spectral Flow Cytometry employed a method described in a previous report 30. In brief, single cells from tumor tissues underwent a 30-min staining with fluorescein-linked monoclonal antibodies at 4 °C and were further detected flow-cytometrically. Specimens were analyzed by FlowJo (version 10.8.1). Monoclonal antibodies included CD45 (Biolegend, cat. No. 103138), CD3 (Biolegend, cat. No. 100210), CD4 (Thermo, cat. No. 48-0041-82), CD8 (BD Pharmingen, cat. No. 557959), CD25 (Biolegend, cat. No. 102008), PD-1 (Biolegend, cat. No. 109118), NK1.1 (Biolegend, cat. No. 108710), CD11b (Biolegend, cat. No. 101228), Gr-1 (Biolegend, cat. No. 108426), CD19 (Biolegend, cat. No. 115543), CD45R/B220 (Biolegend, cat. No. 103244), CD11c (Biolegend, cat. No. 117348), CD86 (Biolegend, cat. No. 105016), CD206 (Biolegend, cat. No. 141720) and MHC-II (Biolegend, cat. No. 107643). The specific flow cytometry analysis strategy is shown in Table 2. Specifically, within the overall FSC and SSC gates, single cells were identified using the FSC-H versus FSC-A plot. Based on empirical criteria, dead cells—typically located in the lower left quadrant—were excluded, and all subsequent data analysis was performed using the same gating strategy.

### Kynurenine treatment and macrophage depletion in mouse models

Control and GPX4-knockdown MFC cells were employed in the mouse study. Mice underwent randomization into six groups. The control and GPX4-knockdown groups were each intraperitoneally injected with 200 μL PBS, 100 mg/kg L-Kynurenine (MCE, cat. No. HY-104026), or 200 μL macrophage depletion agent clodronate liposomes (Encapsula Nano Sciences, cat. No. CLD-8901) every three days, performing body weight measurements. After 15 days of intervention, euthanasia was conducted, followed by tumor excision, imaging and weighing. For the survival model, the animals were similarly assigned to the six aforementioned groups. Body weight was monitored every day. Mice were euthanized when body weight loss exceeded 20% or if the mice exhibited signs of poor health (e.g., inactivity, anorexia, ruffled fur). The date of death was recorded, and survival analysis was subsequently performed.

### Subcutaneous tumor-bearing mouse and CD8 depletion mouse model

The subcutaneous model was established as described above. MFC cells (1×10^5^) were resuspended in 100 µL of PBS and injected subcutaneously into the right flank of the mouse. Subsequently, mice received intraperitoneal injections of 100 µg neutralizing anti-CD8a antibody (BioXcell, cat. No. BE0117) twice per week for a total observation period of 3 weeks. At the end of the 3-week period, the mice were euthanized, and tumors were harvested for photography and weighing.

### Co-culture, conditioned media, and l-kynurenine treatment experiments

Gastric cancer NUGC3 cells, either GPX4-knockdown or control cells, underwent co-culture in Transwell chambers (Corning, cat. No. 3452 and 3464) for 48 h with THP-1 cells (M0 macrophages) pretreated with 320 nM PMA for 6 h. Alternatively, conditioned media from NUGC3 cells (GPX4-knockdown or control) after 48 h of culture were mixed with fresh media at a 1:1 ratio and used to culture PMA-pretreated THP-1 cells (M0) for an additional 48 h. In a third approach, THP-1 cells (M0) pretreated with PMA were further cultured with different concentrations of L-kynurenine (50 μM, 100 μM, 200 μM) for 48 h.

Following these treatments, a portion of the treated THP-1 cells was examined flow-cytometrically to assess changes in CD86 (Biolegend, cat. No. 374216) or CD206 (Biolegend, cat. No. 321106) expression levels. The remaining THP-1 cells were used in subsequent experiments with wild-type NUGC3 cells. Totally 3×10^5^ THP-1 cells underwent seeding into the upper compartment of a Transwell system, with either 5×10^4^ or 1,000 NUGC3 cells seeded in the lower chamber to perform wound healing (0 h and 36 h) and colony formation (2 weeks) assays, respectively. Meanwhile, the Apoptosis kit (Byotime, cat. No. C1062S) was used to assess apoptosis in NUGC3. Additionally, 3×10^5^ co-cultured THP-1 cells were placed in the lower compartment, with 3×10^4^ NUGC3 cells seeded into the superior compartment. After 48 h, tumor cell migration and invasion were assessed. The scratch, clonogenic, and Transwell assays were conducted as previously described 14.

### Metabolomic profiling

Metabolomic profiling was performed on supernatants collected from 2×10^6^ GPX4-knockdown and control NUGC3 cells after 48 h of culture. Metabolites were extracted using 400 μL of chilled methanol/acetonitrile (1:1) for protein precipitation, followed by centrifugation at 14,000 g and 4 °C for 20 minutes, and vacuum-drying. The dry extracts were reconstituted with acetonitrile/water (1:1) and centrifuged again before injection for UHPLC conducted on a 1290 Infinity LC (Agilent) coupled with a QTRAP MS (AB 6500+, AB Sciex). Separation was achieved with HILIC and C18 columns under specific gradient conditions. Mass spectrometry was carried out in the positive and negative ion modes using MRM for quantitative data acquisition. Data were processed using MultiQuant or Analyst software, and metabolites with coefficients of variation (CVs) < 30% in quality control samples were considered reproducible. PCA and OPLS-DA were conducted with SIMCA-P to assess differences between groups. Model robustness was evaluated with 7-fold cross-validation and permutation testing, and P < 0.05 in unpaired Student's t-test was deemed statistically significant.

### Reactive oxygen species assay

To assess reactive oxygen species (ROS) amounts in GPX4-knockdown or overexpression NUGC3 cell models, cells were harvested at 500,000 cells/tube and resuspended in medium containing the DCFH-DA reagent. The cells underwent a 30-minute incubation at 37 °C shielded from light for ROS detection. Following incubation, cells were assessed flow-cytometrically to measure ROS levels based on fluorescence intensity.

### Ferroptosis inducer intervention

NUGC3 cells were seeded at 3×10⁵ cells per well in a 6-well plate and treated with the ferroptosis inducer Erastin (MCE, cat. No HY-15763) (0, 1, and 2 μM) and ferroptosis inhibitor Ferrostatin-1 (MCE, cat. No HY-100579) (0, 0.5, and 1 μM) for 24 h. After treatment, tumor cells were collected for protein expression analysis, and the culture supernatants were collected to measure kynurenine levels.

### Reactive oxygen species assay

To assess reactive oxygen species (ROS) amounts in GPX4-knockdown or overexpression NUGC3 cell models, cells were harvested at 500,000 cells/tube and resuspended in medium containing the DCFH-DA reagent. The cells underwent a 30-minute incubation at 37 °C shielded from light for ROS detection. Following incubation, cells were assessed flow-cytometrically to measure ROS levels based on fluorescence intensity.

### ROS simulation and scavenging assays

In the ROS simulation experiments, NUGC3 cells underwent seeding at 3×10^5^/well in a 6-well plate and treatment with different H_2_O_2_ (0.4 mM, 0.8 mM, 1.2 mM, and 1.6 mM) and 4-HNE (8 nM, 16 nM, and 32 nM) levels for 6 h to induce oxidative stress. After treatment, cells were collected and analyzed for relevant markers by immunoblot and flow cytometry for assessing ROS induction levels.

In the ROS scavenging experiments, cells were treated with the ROS scavenger N-acetylcysteine (NAC) at concentrations of 5 nM and 10 nM, as well as TEMPOL at 5 μM and 10 μM for 24 h. Upon NAC treatment, cell harvest was conducted, followed by immunoblot and flow cytometry to evaluate changes in markers related to ROS production and the effectiveness of ROS scavenging.

### Cytokine and metabolite quantification by ELISA

Cytokines in the peripheral blood of mice were measured using commercial ELISA kits (RayBiotech, cat: QAM-TH17-1-2). In brief, the peripheral blood of mice was collected and placed at room temperature for 30 minutes, followed by centrifugation at 5000 RPM for 10 minutes. The analysis was then performed according to the manufacturer's protocols. The light absorbance values were read at the recommended wavelengths using a microplate reader (Infinite® M1000 Pro).

Cytokine IL-6 levels were quantitated using an ELISA kit (Invitrogen, cat. No. EH2IL6), while kynurenine and tryptophan concentrations were measured with corresponding ELISA kits (Immusmol, cat. No. BA-E-2700 and BA-E-2200). Conditioned media from control and GPX4-knockdown NUGC3 cells were collected after 48 h of culture. Additionally, supernatants from ROS-stimulated or ROS-scavenged samples were collected after 6 or 24 h of intervention, respectively. For intracellular metabolite detection, cells from the same batch were divided into two portions, with one used for protein concentration determination and the other lysed with the appropriate lysis buffer (CST, cat. No. 9803) based on protein concentration to ensure consistency across samples. ELISA assays were then performed on the lysates. Standard curves were generated using standards provided in the various ELISA kits.

For the evaluation of GPX4, kynurenine, and tryptophan levels in fresh clinical samples (collected from Zhejiang Cancer Hospital), the tissues were first minced, followed by lysis. After determining the protein concentration, samples were normalized to ensure equal protein concentrations. A portion of the lysate was employed for subsequent immunoblot to determine GPX4 protein amounts, while the other was assessed with an ELISA kit to measure the levels of corresponding metabolites. Based on the GPX4/housekeeping protein ratio, clinical tissue specimens were categorized into high- and low-GPX4 groups, followed by a correlation analysis with kynurenine/tryptophan expression levels.

### Investigation of KYNU protein degradation mechanism

To explore the degradation mechanisms of the KYNU protein, GPX4-knockdown and control NUGC3 cells were administered MG-132 (5 μM) or BafA1 (10 μM) for a 24-hour incubation. Additionally, wild-type NUGC3 cells underwent pre-treatment with MG132 (5 μM) or BafA1 (10 μM) for 18 h, further co-treated with H_2_O_2_ (0.8 mM) or NAC (10 mM) for 6 h. Cells and proteins were collected for subsequent analysis. For further investigation, GPX4-knockdown and control NUGC3 cells were administered 100 μg/mL CHX for 4, 8, and 12 h, or co-treated with MG-132 (5 μM) or BafA1 (10 μM) for 6 and 12 h. Cells and proteins were collected for downstream assays.

### Co-immunoprecipitation

In GPX4-knockdown and control NUGC3 cells or wild-type NUGC3 cells, lentiviral vectors were used to overexpress exogenous KYNU protein tagged with a Flag epitope. Additionally, plasmids encoding HA-tagged ubiquitin molecules, restricted to either K48 or K63 ubiquitination sites, were co-expressed. These ubiquitin plasmids were purchased from GeneChem (Shanghai, China). After treatment with MG-132 (5 μM), co-immunoprecipitation (Co-IP) was performed using HA (CST, cat. No. 3724S) or Flag (Sigma-Aldrich, cat. No. SAB4200071) antibodies. Co-IP was conducted following the protocol provided with the Classic Magnetic Protein A/G IP/Co-IP Kit (YJ201, Epizyme). Bound proteins were examined by SDS-PAGE and immunoblot to analyze KYNU ubiquitination.

### Site prediction, mutant plasmid construction, and transfection

Potential ubiquitination sites on KYNU were predicted using the PhosphoSitePlus database (http://www.phosphosite.org). The top six lysine residues with the highest ubiquitination scores were selected, and site-directed mutagenesis was performed to replace them with arginine. The resulting mutant plasmids were constructed by GeneChem (Shanghai, China) and transfected into NUGC3 cells. Following transfection, cells underwent a 6-hour treatment with H_2_O_2_ (0.8 mM) or 24-hour treatment with NAC (10 μM). Post-treatment, co-immunoprecipitation and immunoblot were performed to evaluate how these mutations affected KYNU ubiquitination.

For the co culture experiments, conditioned medium from NUGC3 cells (transfected with the aforementioned plasmids and treated with either H₂O₂ or NAC) was used to culture THP1 cells that had been polarized with PMA for 48 h. Subsequently, as described previously, a total of 3×10^5 THP1 cells were seeded into the upper compartment of a Transwell system, while 1,000 NUGC3 cells were seeded into the lower chamber to conduct a colony formation assay over a period of 2 weeks.

### Statistical analysis

Between-group and multiple group comparisons employed the unpaired Student's t-test and one-way analysis of variance (ANOVA), respectively. P < 0.05 was deemed statistically significant.

## Results

### Impacts of GPX4 on immune cell infiltration and GC prognosis

**Figure 1:**
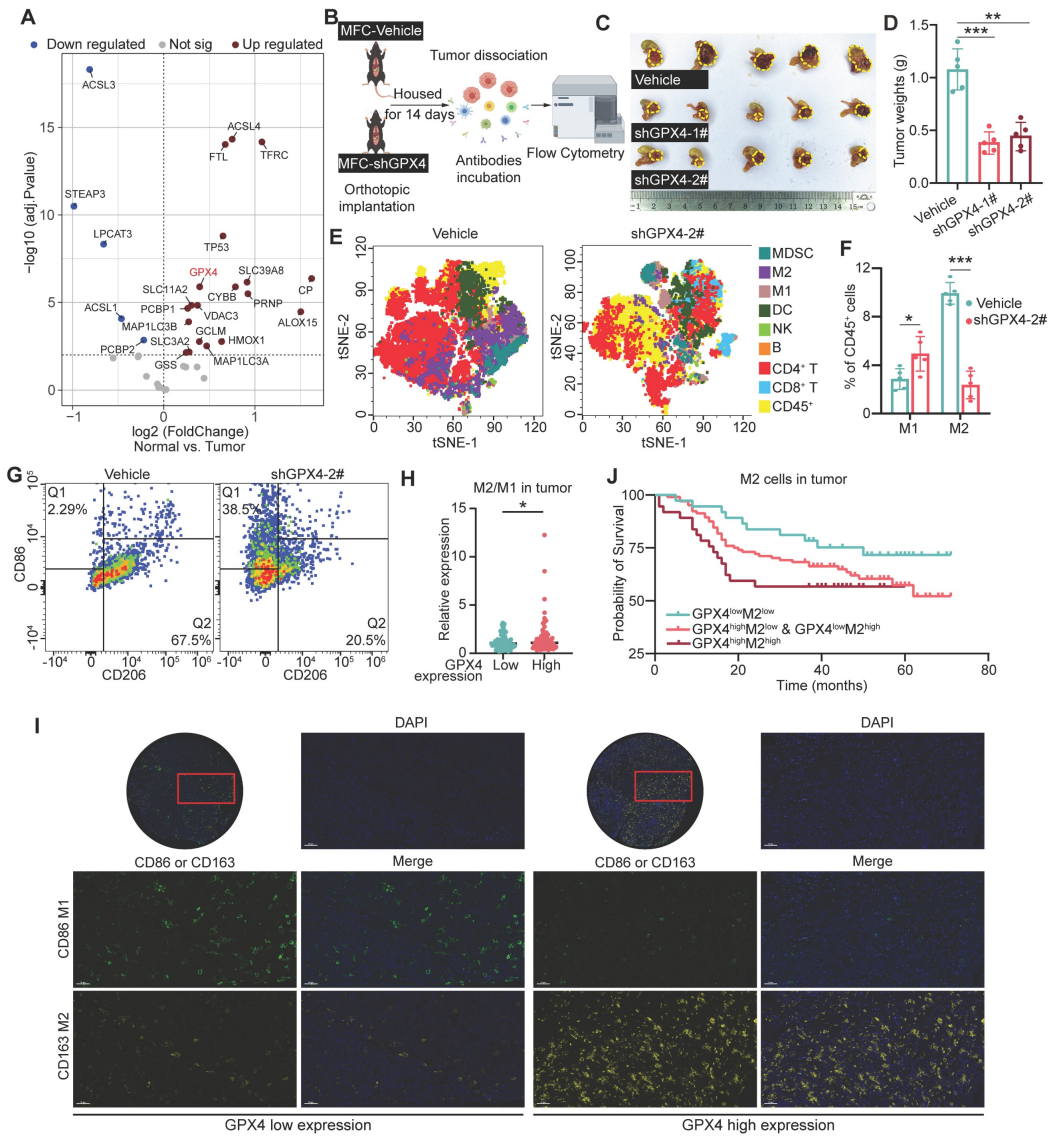
***Impacts of GPX4 knockdown on tumor growth, immune cell infiltration, and patient prognosis in gastric cancer.* (A)** Differential gene expression analysis was performed to assess ferroptosis-related pathways by transcriptomic sequencing of 60 pairs of gastric cancer (GC) and adjacent noncancerous tissue specimens from Zhejiang Cancer Hospital. **(B)** Diagram of the experimental design to assess the effect of GPX4 knockdown on immune cell infiltration in a murine GC cell line (MFC). **(C-D)** Tumor growth (tumor images and weights) in mice was monitored following GPX4 knockdown in MFC cells compared to the control group. **(E)** t-Distributed Stochastic Neighbor Embedding (t-SNE) plots generated from spectral flow cytometry data to visualize immune cell populations. **(F-G)** Impacts of GPX4 knockdown on tumor-associated macrophage (TAM) populations, M1 and M2 macrophages, assessed by spectral flow cytometry. **(H)** A tissue microarray was constructed with tumor samples from 179 GC patients, and multiplex immunohistochemistry was performed to examine the relationship between GPX4 expression and M2/M1 macrophage infiltration ratio within the tumor tissue. **(I)** Representative images of multiplex immunohistochemical staining illustrating the differential infiltration of M1 and M2 macrophages in tumor tissue samples with varying GPX4 expression levels. Bar = 50 μm **(J)** Kaplan-Meier survival analysis conducted to evaluate the prognostic significance of GPX4 expression and macrophage infiltration.

Totally 60 pairs of GC and adjacent noncancerous tissue samples were obtained from Zhejiang Cancer Hospital for transcriptomics. Differential gene analysis of ferroptosis-related pathways revealed elevated expression of genes, including GPX4, ACSL4, TFRC, and ALOX15, in GC tissues **(Figure 1A and Figure S1A)**. In the MFC cell-derived subcutaneous xenograft model, GPX4-knockdown reduced tumor volume compared to scramble controls (P < 0.001), demonstrating potent suppression of gastric cancer progression (Figure 1B-D and Figure S1B). Flow cytometry demonstrated that GPX4-knockdown reshaped the GC immune landscape, with CD8^+^ T cells and M1 macrophages increased, concomitant with reduction in CD4^+^ T cells and M2 macrophages (Figure 1E-G and Figure S1C). Furthermore, after GPX4 knockdown, peripheral blood from the mice showed a significant increase in IL-1β, IL-6, IL-12, IFN-γ, and TNF-α, which are associated with macrophage immune activation, while IL-10 and TGF-β, which are associated with anti-inflammatory responses, significantly decreased **(Figure S1D)**. This suggests that GPx4 plays an important role in regulating the immune microenvironment of GC.

Next, we explored the relationship between macrophages and GPX4 expression using tissue microarray analysis. The results showed that GPX4 expression primarily influences macrophage infiltration within the tumor, but not in the stroma **(Figure 1H-I and Figure S1E-G)**. The M2/M1 ratio in the GPX4-high group was higher than that in the GPX4-low group **(Figure 1H-I)**. Notably, the GPX4-high group exhibited reduced M1 macrophage infiltration (P = 0.026), while M2 infiltration did not differ significantly (P = 0.496) **(Figure S1G)**. Survival analysis showed that the 5-year overall survival (OS) rate in GPX4^low^M2^low^ group was higher than that in GPX4^high^M2^high^ group (73.0% vs 56.8%, P = 0.048). In contrast, while there was no statistical significance in 5-year OS between GPX4^low^M1^high^ group and GPX4^high^M1^low^ group (P = 0.113), GPX4^low^M1^high^ group also showed higher 5-year OS (71.4% vs 54.7%). This suggests that GPX4 overexpression dominance synergizes with macrophages to drive poor outcomes.

### GPX4 knockdown in GC cells suppresses tumor growth by modulating macrophage polarization

**Figure 2:**
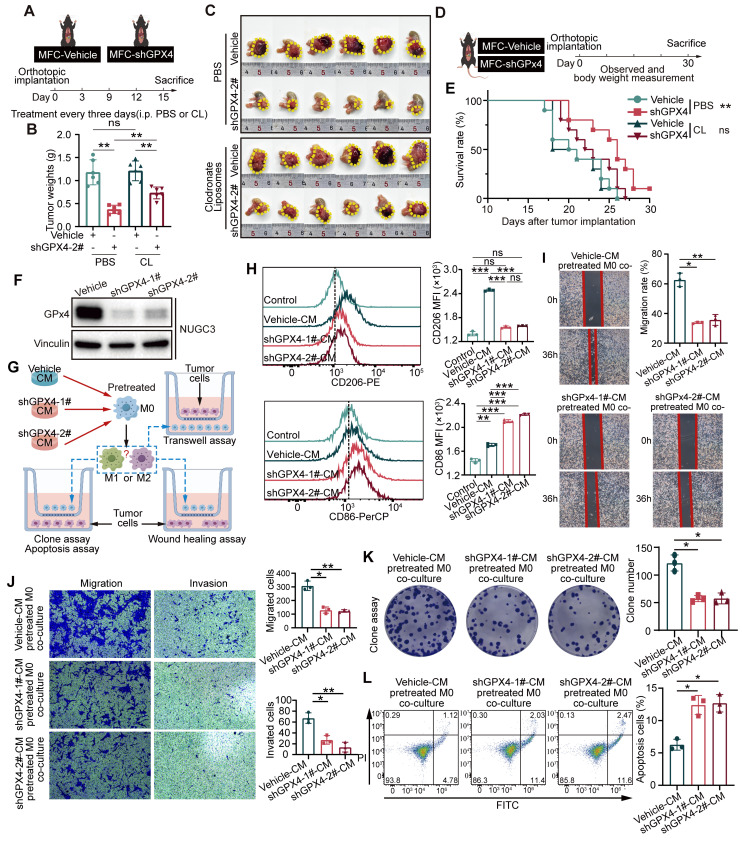
***GPX4 knockdown modulates macrophage polarization and suppresses tumor growth in gastric cancer.* (A)** Schematic representation of macrophage depletion using Clodronate Liposomes (CL) in mice to evaluate the role of macrophages in GPX4 knockdown-induced tumor suppression. **(B-C)** Experimental setup to evaluate the effects of GPX4 knockdown combined with macrophage depletion on tumor growth, as assessed by tumor weight and representative images, in an orthotopic gastric cancer (GC) mouse model. **(D-E)** Design of the orthotopic mouse model used to assess the survival benefits of GPX4 knockdown and the impacts of macrophage depletion on survival outcomes. **(F)** Western blot analysis confirming the efficiency of GPX4 knockdown in the human gastric cancer cell line (NUGC3). **(G)** Schematic diagram of a design for investigating macrophage polarization changes and their functional impact. THP-1 cells, pretreated with 320 nM PMA for 6 h, were cultured with conditioned media from either vehicle or GPX4-knockdown GC cells for 48 h. **(H)** Flow cytometry analysis of macrophages following pretreatment with NUGC3 cell-conditioned medium. **(I-L)** Assays of NUGC3 cells co-cultured with macrophages including wound-healing assay** (I)**, Transwell migration assay **(J)**, colony formation assay **(K)** and apoptosis assay** (L)**.

To elucidate the role played by macrophage polarization changes in the effect of GPX4 knockdown on GC cells, macrophages were depleted in mice with Clodronate Liposomes (CL)** (Figure 2A)**. The results indicated that macrophage depletion attenuated the tumor-suppressive effects of GPX4 knockdown **(Figure 2B-C)**. Specifically, under macrophage depletion conditions, the tumor mass in the GPX4 knockdown group was restored to 61.99% of the control group, compared to 31.62% in the non-depleted group (P < 0.01), indicating that macrophages mediated approximately 30.37% of the GPX4-dependent tumor-suppressive effects. In a parallel experiment, rescue experiments using CD8⁺ T cell depletion demonstrated that CD8⁺ T cells contributed only 17.26% of the anti-tumor effect **(Figure S2A)** (P < 0.05), highlighting the more significant role of macrophages. Additionally, in an orthotopic mouse model of tumors, macrophage depletion similarly reduced the survival benefit conferred by GPX4 knockdown **(Figure 2D-E)**. Wound-healing, colony formation, and transwell assays revealed that monocytes (THP-1) co-cultured with GPX4-knockdown NUGC3 cells markedly suppressed migration, proliferation, and invasion in GC cells **(Figure S2C-E)**. We further investigated whether GPX4 expression in tumor cells affects macrophage polarization and, consequently, tumor cell behavior **(Figure 2F-G and Figure S2B)**.

In particular, the lL-10 and TGF-β secreted by macrophages were reduced, while TNF-α was increased after co-cultured with GPX4-knockdown NUGC3 cells. These changes were accompanied by an upregulation of CD86 and a downregulation of CD206 on the macrophage surface, compared to co-culture with control NUGC3 cells **(Figure S2F-G)**. Moreover, conditioned medium from GC cells produced similar results **(Figure 2H and Figure S2H)**. Both GPX4 knockdown NUGC3 cells and their corresponding conditioned medium inhibited the mRNA levels of angiogenesis-related factors VEGFA and MMP9 in THP-1-derived macrophages **(Figure S2F and S2H)**. Additional functional assays indicated that GPX4 expression in GC cells affected macrophage-mediated tumor cell killing through changes in conditioned media **(Figure 2I-L)**.

These findings indicate that alterations in GPX4 expression in tumor cells modulate both macrophage polarization and CD8⁺ T cell infiltration, with macrophages playing a more pivotal role. Moreover, conditioned media from GPX4-knockdown tumor cells demonstrated a similar ability to control macrophage polarization and cytokine secretion, indicating that tumor cells may regulate macrophage polarization through the secretion of distinct metabolites or secreted proteins such as cytokines and chemokines.

### GPX4 silencing in GC cells regulates macrophage polarization by affecting kynurenine levels

**Figure 3:**
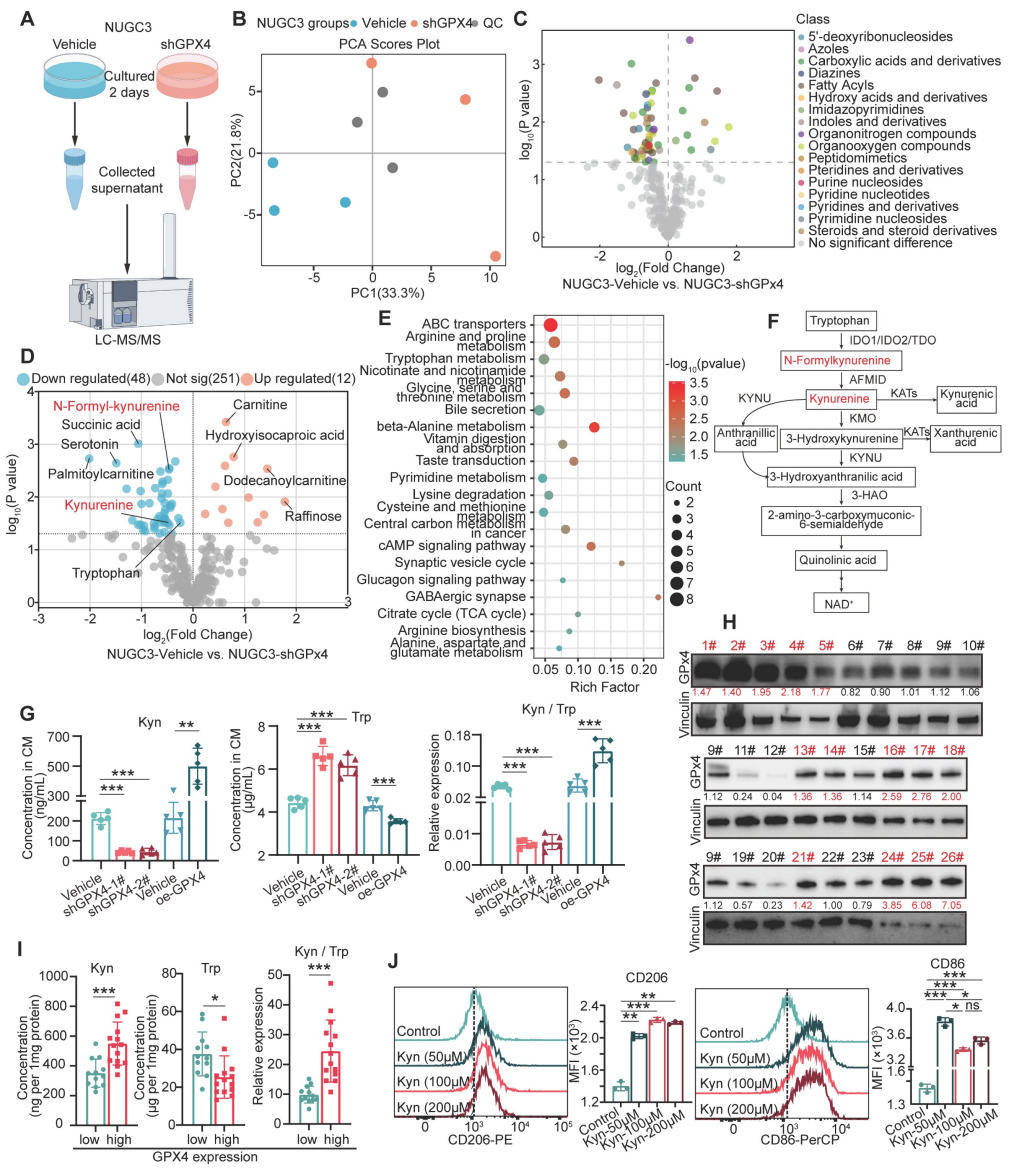
***Metabolomic analysis of the impacts of GPX4 knockdown on metabolite levels and macrophage polarization.* (A)** Schematic representation of the metabolomic analysis workflow, including sample collection and the mass spectrometry procedure.** (B)** Quality control of metabolomic data, presented as principal component analysis (PCA) plots. **(C)** Volcano plot depicting differential metabolite families, including 5'-deoxyribonucleosides and azole carboxylic acids and derivatives, between vehicle and GPX4-knockdown NUGC3 cells.** (D)** Volcano plot highlighting differential metabolites, with a focus on N-methyl-kynurenine and kynurenine (kyn), in GPX4-knockdown NUGC3 cells.** (E)** Pathway enrichment analysis revealing significant enrichment in the tryptophan (Trp) metabolism pathway in GPX4-knockdown NUGC3 cells.** (F)** Flowchart of tryptophan and kynurenine metabolism. **(G)** Kynurenine levels, tryptophan levels, and the kynurenine/tryptophan ratio in the supernatant of GPX4-knockdown NUGC3 cells.** (H-I)** GPX4 expression and kynurenine levels in fresh gastric cancer tissues from 26 patients, including kynurenine and tryptophan measurements.** (J)** Flow cytometry analysis of macrophage polarization (M1 and M2) in response to varying kynurenine concentrations (50, 100, and 200 μM for 48 h) using THP-1 cells.

To explore the mechanism of GPx4 regulation of macrophage polarization, metabolomic analyses of supernatants from control and GPX4-knockdown gastric cancer cells were performed **(Figure 3A-B)**. Our results indicated that GPX4 knockdown significantly altered the secretion of several groups of metabolites, including 5'-deoxyribonucleosides and azoles carboxylic acids and derivatives **(Figure 3C)**. Notably, N-methyl-kynurenine and kynurenine (kyn) amounts were markedly decreased in the supernatants of GPX4-knockdown cells** (Figure 3D)**, with pathway enrichment analysis highlighting a significant enrichment in the tryptophan metabolism pathway **(Figure 3E-F)**. Previous studies have demonstrated that abnormal tryptophan (Trp) metabolism promotes tumor progression by suppressing the cytotoxic activities of multiple immune cells, e.g., CD4^+^ and CD8^+^ T cells 31. We further confirmed the reduction in kynurenine amounts and lower kynurenine/tryptophan (Kyn/Trp) ratios in both the supernatants and lysates of GPX4-knockdown gastric cancer cells **(Figure 3G)**. Moreover, tumor tissues from tumor-bearing mice also exhibited low kynurenine levels following GPX4 knockdown **(Figure S3A)**. Fresh gastric cancer samples from 26 patients at Zhejiang Cancer Hospital were collected, and GPX4 expression and the metabolic levels of kynurenine and tryptophan in the corresponding tissues were analyzed. As depicted in **Figure 3H-I**, tissues with high GPX4 expression displayed elevated kynurenine and reduced tryptophan amounts. Next, THP-1 cells were administered varying kynurenine amounts. **Figure 3J** shows that high concentrations of kynurenine promoted M2 polarization in macrophages and inhibited M1 polarization. Additionally, macrophages pretreated with various kynurenine amounts were co-cultured with GC cells. The results demonstrated that kynurenine-treated macrophages enhanced migration, invasion, and proliferation in GC cells concentration-dependently **(Figure S3B-E)**. Further animal experiments showed that intraperitoneal injection of kynurenine in mice reduced the tumor-suppressive effects and survival benefits associated with GPX4 knockdown in GC cells **(Figure S3F-H)**. Overall, these findings suggest that GPX4 knockdown in tumor cells may inhibit macrophage M2 polarization by lowering kynurenine levels in the cellular environment.

### GPX4 regulates kynurenine metabolism-related enzymes by modulating ros levels in GC

**Figure 4:**
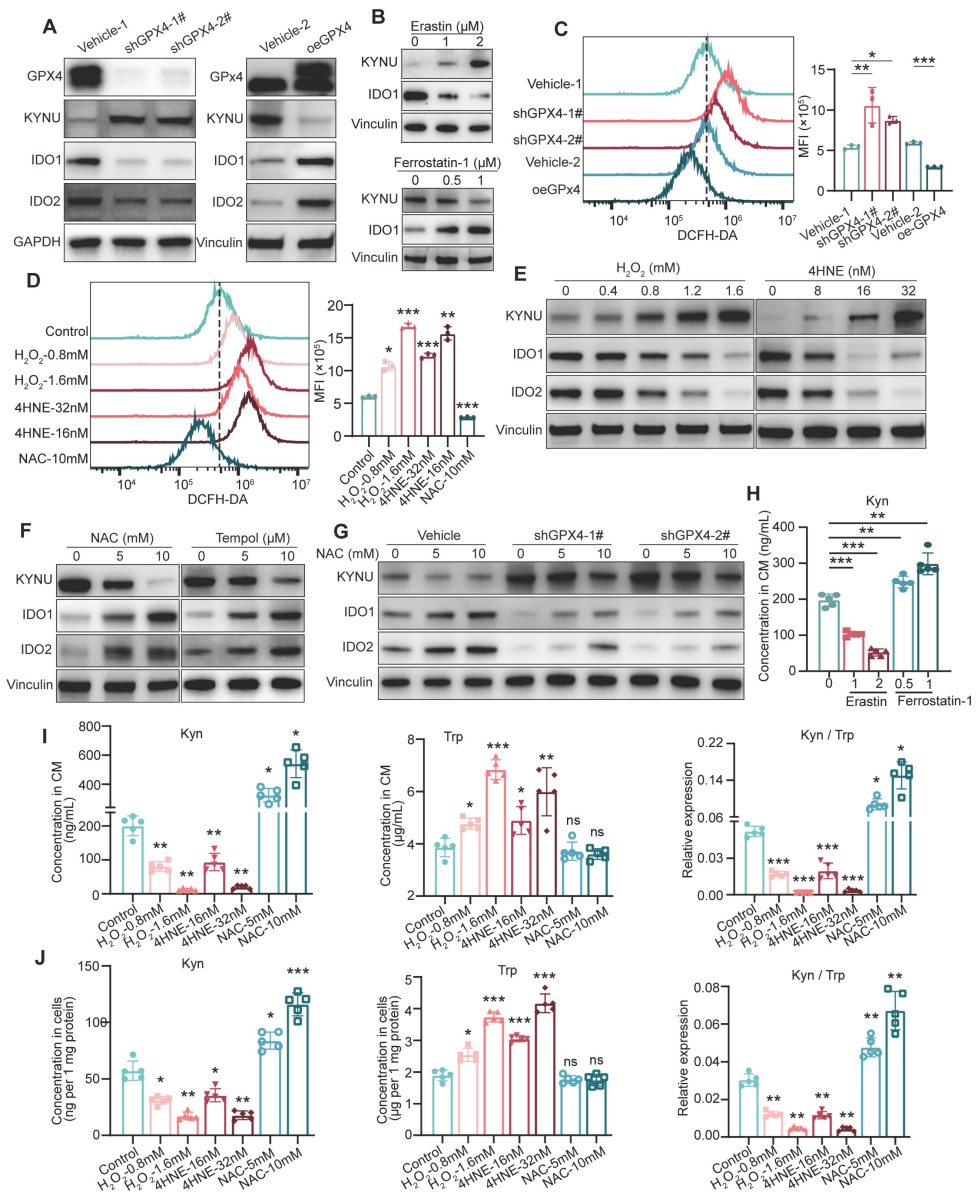
***Regulation of kynurenine metabolism by GPX4 through modulation of intracellular ROS levels.* (A)** Western blot analysis of kynurenine metabolism-related enzymes (KYNU, IDO1, and IDO2) in NUGC3 with GPX4 knockdown or overexpression. **(B)** KYNU and IDO1 expressions in NUGC3 cells after Erastin (1 and 2 μM) and Ferrostatin-1(0.5 and 1 μM) treatment. **(C-D)** Intracellular ROS levels measured by flow cytometry to evaluate the impacts of these treatments on ROS levels in NUGC3 cells with GPX4 knockdown or overexpression, or treated with hydrogen peroxide (H_2_O_2_) and 4-hydroxy-2-nonenal (4HNE) for 6 h, or N-acetylcysteine (NAC) for 24 h. **(E)** Dose-dependent effects of H_2_O_2_ and 4HNE on KYNU, IDO1, and IDO2 expression in NUGC3 cells.** (F)** Dose-dependent effects of NAC and TEMPOL on KYNU, IDO1, and IDO2 expression in NUGC3 cells.** (G)** Effects of ROS scavenging (via NAC) on KYNU, IDO1, and IDO2 expression in cells with GPX4 knockdown. **(H)** Kynurenine levels in NUGC3 cell culture supernatants after Erastin and Ferrostatin-1 treatment. **(I-J)** Kynurenine and tryptophan levels in cell culture supernatants and lysates, along with the kynurenine/tryptophan (Kyn/Trp) ratios, following ROS modulation by H_2_O_2_, 4HNE, or NAC.

To investigate how GPX4 affects kynurenine metabolism, the expression of kynurenine metabolism-related enzymes, including KYNU, IDO1, and IDO2, were measured in GC cells. In this study, GPX4 knockdown or knockout starkly increased the levels of KYNU, which transforms kynurenine, while the levels of IDO1 and IDO2, which produce kynurenine, were significantly reduced. Conversely, overexpression of GPX4 exerted the opposite effects **(Figure 4A and Figure S4A)**. Additionally, we treated tumor cells with the ferroptosis activator erastin and observed that erastin significantly increased KYNU expression while suppressing IDO1 expression; in contrast, the ferroptosis inhibitor Ferrostatin-1 produced the opposite result** (Figure 4B)**. Given that GPX4 expression significantly affects intracellular ROS levels, we hypothesized that GPX4 regulates kynurenine-related metabolic enzymes by modulating ROS levels. First, we found that GPX4 knockdown remarkably increased intracellular ROS levels, whereas its overexpression had the opposite effects **(Figure 4C)**. Additionally, hydrogen peroxide (H_2_O_2_) and 4-hydroxy-2-nonenal (4HNE) starkly elevated intracellular ROS levels, while N-acetylcysteine (NAC) effectively reduced ROS levels** (Figure 4D)**. The results showed that H_2_O_2_ and 4HNE increased KYNU expression in a dose-dependently, while decreasing IDO1 and IDO2 amounts **(Figure 4E and Figure S4C)**. Conversely, NAC intervention reduced KYNU expression and increased IDO1 and IDO2 levels. A similar outcome was observed with another ROS scavenger, TEMPOL **(Figure 4F)**. Importantly, ROS scavenging significantly mitigated the increase in KYNU and decreases in IDO1 and IDO2 induced by GPX4 knockdown **(Figure 4G)**. We also observed that treating GC cells with Erastin significantly reduced kynurenine levels in the culture supernatant, while treatment with Ferrostatin-1 led to a marked increase in kynurenine levels **(Figure 4H)**. Furthermore, H_2_O_2_ and 4HNE both reduced the levels of intracellular and secreted kynurenine dose-dependently, decreased the kynurenine/tryptophan ratio, and increased tryptophan levels **(Figure 4I-J and Figure S4D)**. Rescue experiments with NAC and H_2_O_2_ in GPX4-knockdown and -overexpressing GC cells showed that NAC elevated kynurenine levels and inhibited the increase in tryptophan in GPX4-knockdown cells; H_2_O_2_ impaired the regulation of kynurenine and tryptophan by GPX4 overexpression **(Figure S4E-F)**. In macrophage polarization assays with conditioned medium, NAC reversed the effects of GPX4 knockdown on M1 and M2 polarization, while H_2_O_2_ disrupted the effects of GPX4 overexpression on M1 and M2 polarization** (Figure S4G-H)**. In summary, GPX4 regulates kynurenine metabolism-related enzymes by controlling intracellular ROS levels.

### The reduction in kynurenine induced by GPX4 Knockdown in GC cells is due to the upregulation of KYNU

**Figure 5:**
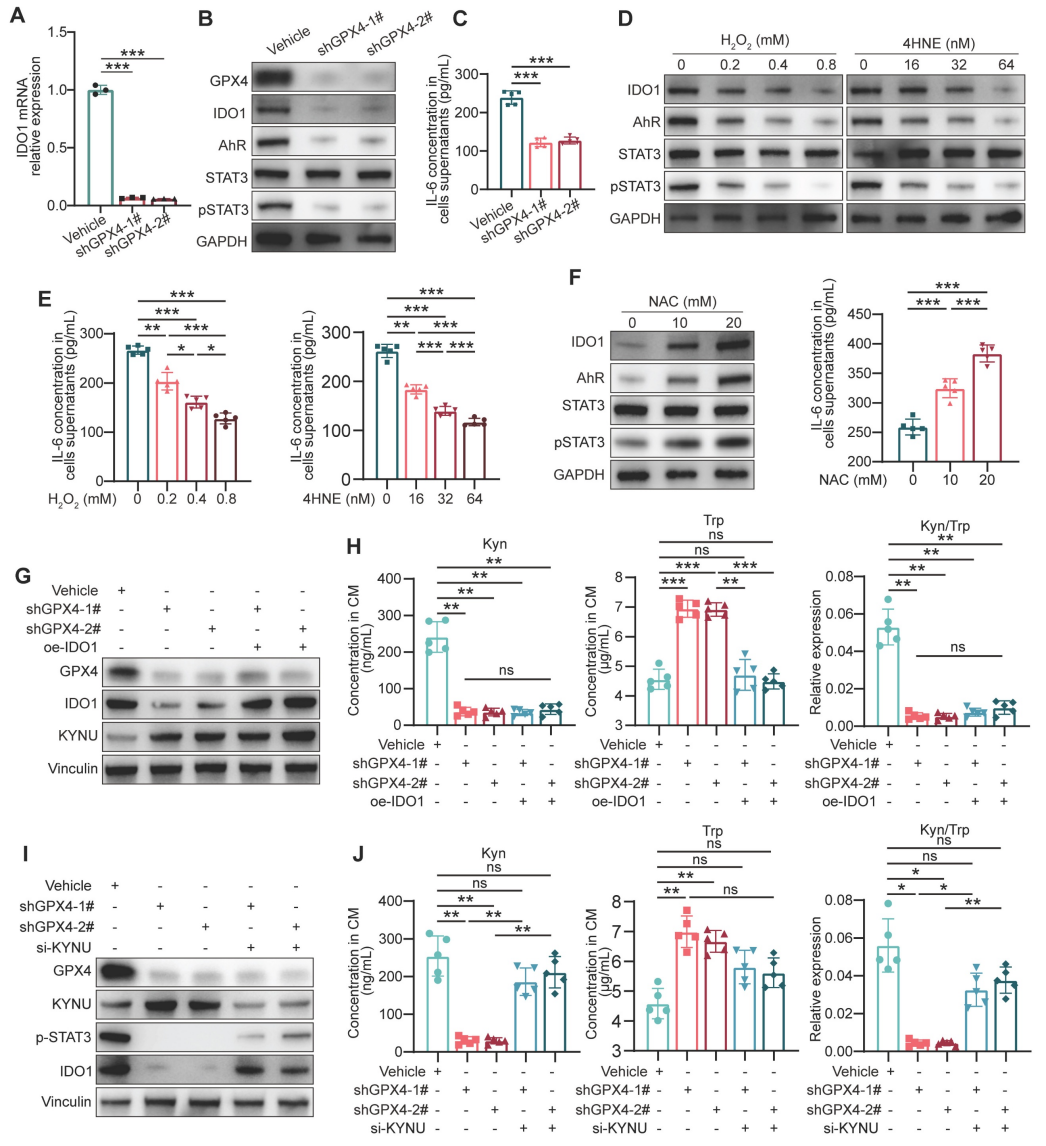
***Regulation of IDO1 expression and kynurenine metabolism by GPX4 knockdown in gastric cancer cells.* (A)** Quantitative PCR analysis of the effect of GPX4 knockdown on IDO1 mRNA levels in NUGC3 cells. **(B-C)** Western blot analysis of the impacts of GPX4 knockdown on the expression of AhR, IL-6, and p-STAT3. **(D-E)** Effects of exogenous ROS on the expression of AhR, IL-6, and p-STAT3, as assessed by Western blotting.** (F)** Impacts of ROS scavenging (NAC treatment) on the expression of AhR, IL-6, and p-STAT3. **(G)** Western blot analysis of GPX4, IDO1, and KYNU protein expression levels to verify overexpression efficiency. **(H)** Quantification of kynurenine, tryptophan, and kynurenine/tryptophan (Kyn/Trp) ratio in the cell lines described in the panel. **(I)** Western blot analysis of protein expression levels of GPX4, KYNU, p-STAT3, and IDO1 to verify knockdown efficiency and regulation.** (J)** Quantification of kynurenine, tryptophan, and the kynurenine/tryptophan ratio in the cell lines described in the panel.

Since IDO1 is crucial for kynurenine metabolism in tumor cells, how GPX4 regulates IDO1 levels via ROS was first investigated. qPCR revealed that GPX4 knockdown in gastric cancer cells led to a corresponding decrease in IDO1 mRNA levels **(Figure 5A)**. Previously reported data suggest kynurenine in cancer cells activates IDO1 transcription through the AhR/IL-6/STAT3 pathway in a positive feedback loop 32. Expectedly, GPX4 silencing starkly downregulated AhR, IL-6, and p-STAT3** (Figure 5B-C)**. Further experiments demonstrated that exogenous ROS substantially downregulated AhR, IL-6, and p-STAT3 **(Figure 5D-E)**, while ROS scavenging significantly stimulated their expression **(Figure 5F)**. To confirm the importance of IDO1 expression in kynurenine metabolism, a series of rescue experiments were conducted. As depicted in** Figure 5G-H**, IDO1 overexpression failed to rescue the kynurenine reduction induced by GPX4 knockdown and did not affect KYNU expression. Therefore, we hypothesized that KYNU might be the primary factor altering kynurenine metabolism due to GPX4 knockdown. Further experiments showed that inhibiting KYNU upregulation using siRNA significantly restored the reduced IDO1 expression and kynurenine levels caused by GPX4 knockdown **(Figure 5I-J)**. Taken together, the reduction in kynurenine induced by GPX4 knockdown is likely due to KYNU upregulation. The downregulated kynurenine affects IDO1 expression through AhR/IL-6/STAT3 signaling but is not the primary cause of the observed changes in kynurenine levels.

### GPX4 knockdown affects KYNU protein stability by converting its ubiquitination from Ub-K48 to Ub-K63

**Figure 6:**
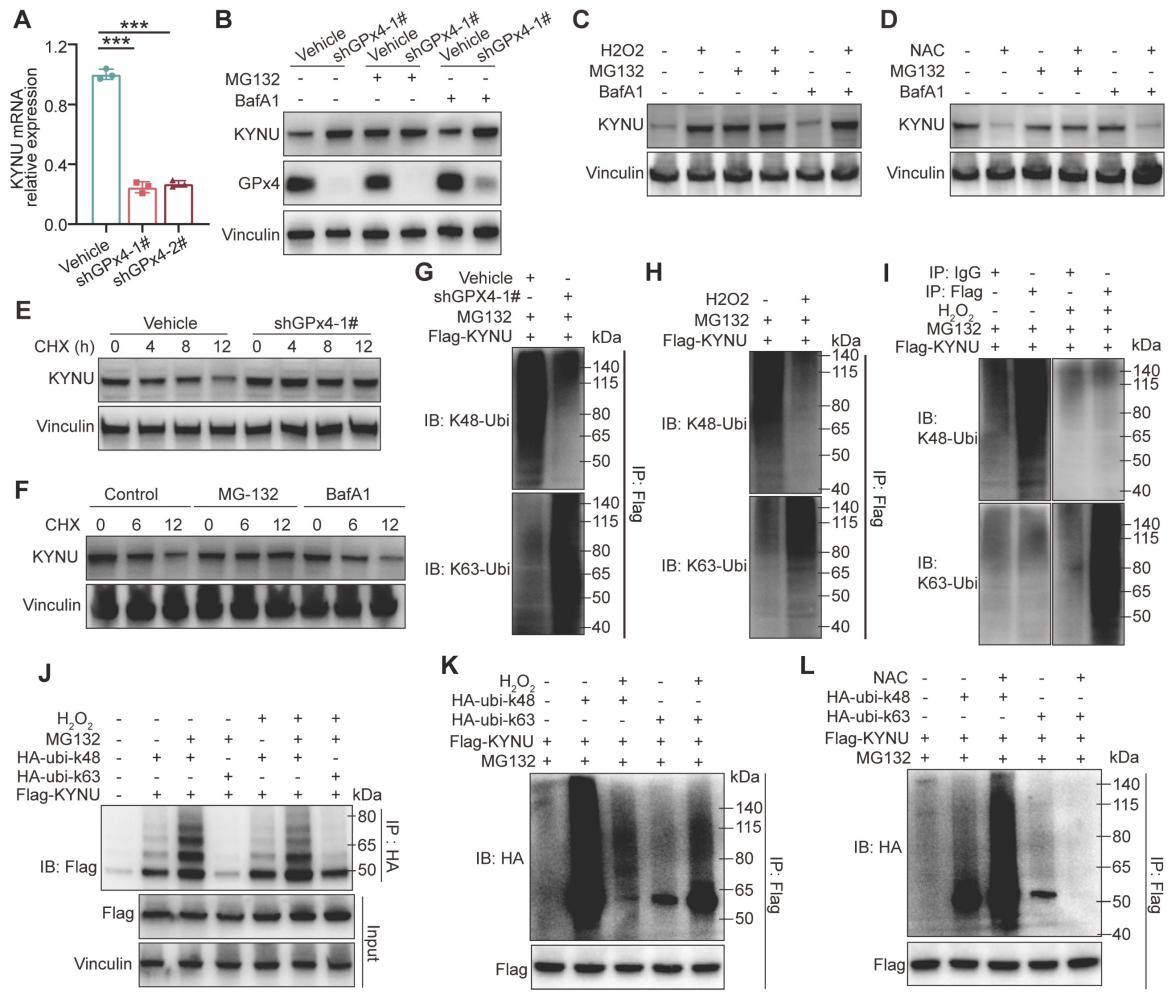
***GPX4 knockdown regulates KYNU protein stability by altering ubiquitination patterns.* (A)** Quantitative PCR analysis of KYNU mRNA levels in NUGC3 cells following GPX4 knockdown. **(B)** Western blot analysis of KYNU protein expression in vehicle and GPX4-knockdown cells treated with the proteasome inhibitor MG132 (5 μM, 24 h) or the autophagy inhibitor BafA1 (10 μM, 24 h). **(C-D)** Degradation pathways of the KYNU protein in NUGC3 cells pre-treated with MG132 (5 μM) or BafA1 (10 μM) for 18 h, further co-treated with H_2_O_2_ (0.8 mM) or NAC (10 mM) for 6 h. **(E)** KYNU protein degradation over time, analyzed by western blot, in control and GPX4-knockdown NUGC3 cells treated with cycloheximide (CHX, 100 ng/mL) for 0, 4, 8, and 12 h. **(F)** Western blot analysis of pathways involved in KYNU degradation in NUGC3 cells treated with CHX (100 ng/mL) in combination with MG132 (5 μM) or BafA1 (10 μM) for 0, 6, and 12 h. Immunoprecipitation assays were conducted in **(G-L)**. **(G)** In NUGC3 cells with GPX4 knockdown or transfected with the control vector, Flag-KYNU was overexpressed and treated with MG132 (5 μM) for 24 h. **(H-I)** In NUGC3 cells overexpressing Flag-KYNU, cells were treated with MG132 (5 μM) for 24 h. **(J)** NUGC3 cells overexpressing Flag-KYNU were also transfected with HA-ubi-K48 or HA-ubi-K63 constructs and treated with H_2_O_2_ (0.8 mM) and/or MG132 (5 μM). **(K-L)** In NUGC3 cells overexpressing Flag-KYNU and HA-ubi-K48 or HA-ubi-K63, cells were treated with H_2_O_2_ (0.8 mM) or NAC (10 mM) in combination with MG132 (5 μM).

Next, we investigated how GPX4 regulates KYNU protein expression through ROS. Rt-qPCR analysis showed that GPX4 knockdown reduced KYNU mRNA levels **(Figure 6A)**, suggesting that the increase in KYNU protein observed following GPX4 knockdown is unlikely to result from enhanced transcriptional regulation, but is more likely due to increased protein stability. Upon treatment with the proteasome suppressor MG132 and the autophagy suppressor BafA1 in both vehicle and shGPX4 groups, KYNU protein levels in the vehicle group treated with MG132 were similar to those of the knockdown and other drug-treated knockdown groups. In contrast, KYNU protein levels in the vehicle group treated with BafA1 remained low and were similar to those of the untreated group **(Figure 6B)**. These results were consistent with those obtained with exogenous ROS **(Figure 6C)**. Additionally, MG132 successfully prevented NAC-induced KYNU degradation **(Figure 6D)**. These findings suggest ROS elevation by GPX4 silencing inhibits proteasomal degradation of KYNU, while ROS scavenging promotes proteasomal degradation of KYNU. This hypothesis was confirmed by cycloheximide (CHX) experiments **(Figure 6E-F)**. Proteasomal degradation of target proteins typically requires ubiquitination, with K48-linked ubiquitination primarily mediating protein degradation and K63-linked ubiquitination primarily potentiating protein function. For example, the ubiquitin ligase TRAF6 promotes K63-linked ubiquitination of ULK1, thereby enhancing its stability and function, which plays an important role in autophagy induction 33,34. Our results indicated that, under normal conditions, KYNU was predominantly modified by Ub-K48 rather than Ub-K63. Addition of exogenous ROS altered this modification pattern, causing KYNU to be primarily modified by Ub-K63 instead of Ub-K48 **(Figure 6G-K)**. Conversely, ROS scavenging (NAC treatment) further enhanced Ub-K48 and reduced Ub-K63 modification of KYNU. These results indicate that the KYNU protein is normally modified by Ub-K48 and degraded via the proteasomal pathway. However, GPX4 knockdown increases ROS levels, which converts Ub-K48 modification to Ub-K63 modification, thereby inhibiting the degradation of KYNU and increasing the stability of KYNU protein levels.

### Specific ubiquitination sites of the KYNU protein

**Figure 7:**
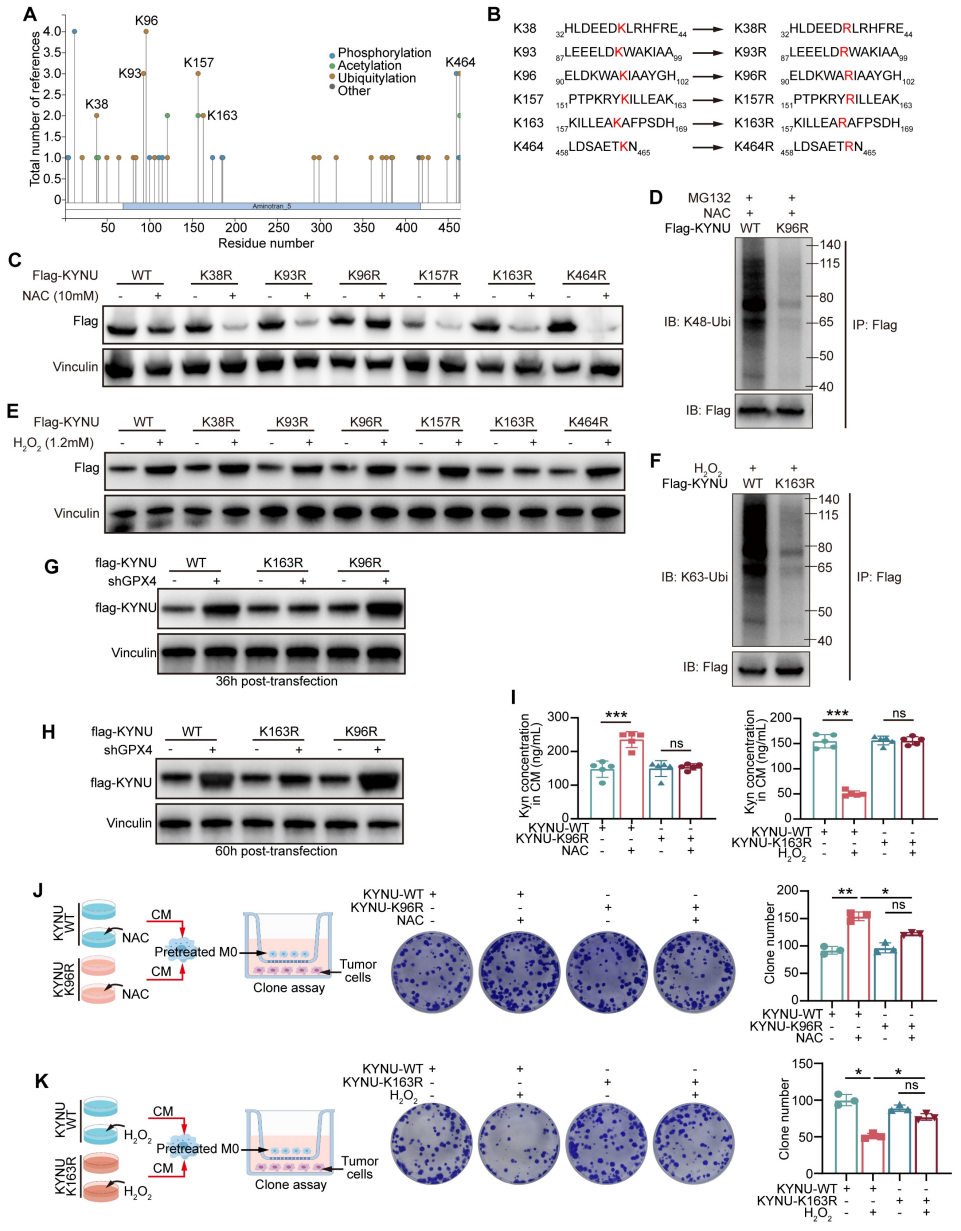
***Identification and functional analysis of the key ubiquitination sites of the KYNU protein modulated by ROS.* (A)** Potential ubiquitination sites of the KYNU protein were identified using http://www.phosphosite.org. **(B)** Six frequently modified lysine (K) residues of KYNU were selected from the literature and mutated to arginine (R). **(C)** Western blot analysis of KYNU stability in NUGC3 cells overexpressing wild-type or lysine-mutant KYNU (K96R, K163R, and other mutants) following treatment with NAC (10 mM) for 24 h. Results indicate that K96R mutation prevents NAC-induced degradation of KYNU. **(D)** Co-immunoprecipitation analysis of K48-ubiquitinated KYNU in NUGC3 cells overexpressing wild-type KYNU or K96R-KYNU after a 24-hour treatment with MG132 (5 μM) and NAC (10 mM). Results show a significant reduction in K48-ubiquitination in the K96R mutant. **(E)** Western blot analysis of KYNU stability in NUGC3 cells overexpressing wild-type or lysine-mutant KYNU (K96R, K163R, and other mutants) after treatment with H_2_O_2_ (10 mM) for 6 h. The K163R mutation prevents ROS-induced stabilization of KYNU. **(F)** Co-immunoprecipitation analysis of K63-ubiquitinated KYNU in NUGC3 cells overexpressing wild-type KYNU or K163R-KYNU following pre-treatment with MG132 (5 μM) for 18 h and further co-treatment with H_2_O_2_ (10 mM) for 6 h. The results indicate that the K163R mutation reduces K63-ubiquitination of KYNU. **(G-H)** Expression of flag-KYNU in WT-, K163R-, and K96R-KYNU plasmid-transfected control and GPX4 knockdown NUGC3 gastric cancer cells at 36- and 60-hour post-transfection. **(I)** Kynurenine levels in cells with KYNU-K96R or KYNU-K163R mutations under NAC or H_2_O_2_ conditions to evaluate the functional effects on kynurenine metabolism. **(J-K)** Design of the co-culture strategy and colony formation assay in NUGC3 cells overexpressing wild-type KYNU, K96R-KYNU, or K163R-KYNU after treatment with H_2_O_2_ for 6 h or NAC for 24 h, showing differential effects on cell growth.

To further identify the specific ubiquitination sites of KYNU, we retrieved the potential ubiquitination sites listed on http://www.phosphosite.org **(Figure 7A)**. Then, the top six most frequently modified lysine (K) residues based on the literature were selected and mutated to arginine (R) residues **(Figure 7B)**. The results showed that after mutating the K96 site of KYNU, ROS scavenging (NAC intervention) no longer promoted its degradation **(Figure 7C)**, and the Ub-K48 ubiquitination of KYNU was significantly reduced **(Figure 7D)**. These findings indicate that the K96 site is the primary site for Ub-K48 ubiquitination under normal or low ROS conditions. Furthermore, when the K163 site of KYNU was mutated, addition of ROS did not increase KYNU stability **(Figure 7E)**, and the Ub-K63 ubiquitination of KYNU was significantly reduced **(Figure 7F)**.

The above data suggest that K163 is the primary site for Ub-K63 ubiquitination under high ROS conditions, and that this modification may enhance the stability of the KYNU protein. In experiments with GPX4-knockdown cells, WT-KYNU expression increased significantly at 36 h post-transfection, with a similar effect on K96R-KYNU, while K163R-KYNU showed no significant increase **(Figure 7G)**. At 60 h, K163R-KYNU exhibited a slight increase, but lower than WT-KYNU, while K96R-KYNU showed a significant increase in shGPX4 cells** (Figure 7H)**. This suggests that K163R-KYNU's lack of K63-linked ubiquitination at 36 h affects its stability, while at 60 h, K96R-KYNU's inability to undergo K48-linked ubiquitination contributed to increased expression. These results align with previous findings.

Next, the effects of these site-specific mutations on kynurenine metabolism were examined. The results showed that the K96 mutation of KYNU blocked the NAC-induced increase of kynurenine levels, while K163 mutation impaired the ROS-mediated reduction of kynurenine levels **(Figure 7I)**. According to the co-culture strategy in** Figure 7J-K**, NUGC3 cells transfected with WT-, K96R-, and K163R-KYNU were treated with NAC or H2O2, and the conditioned media were used to culture macrophages. These macrophages were then co-cultured with fresh tumor cells, and tumor cell proliferation was assessed by colony formation assay. The results showed that lysine mutations at K96 and K163 impaired the functions of the corresponding ROS regulators. In summary, the K96 site of KYNU is the primary site for promoting Ub-K48 ubiquitination under low ROS conditions, while the K163 site is the primary site for promoting Ub-K63 ubiquitination in the presence of ROS.

## Discussion

**Figure 8:**
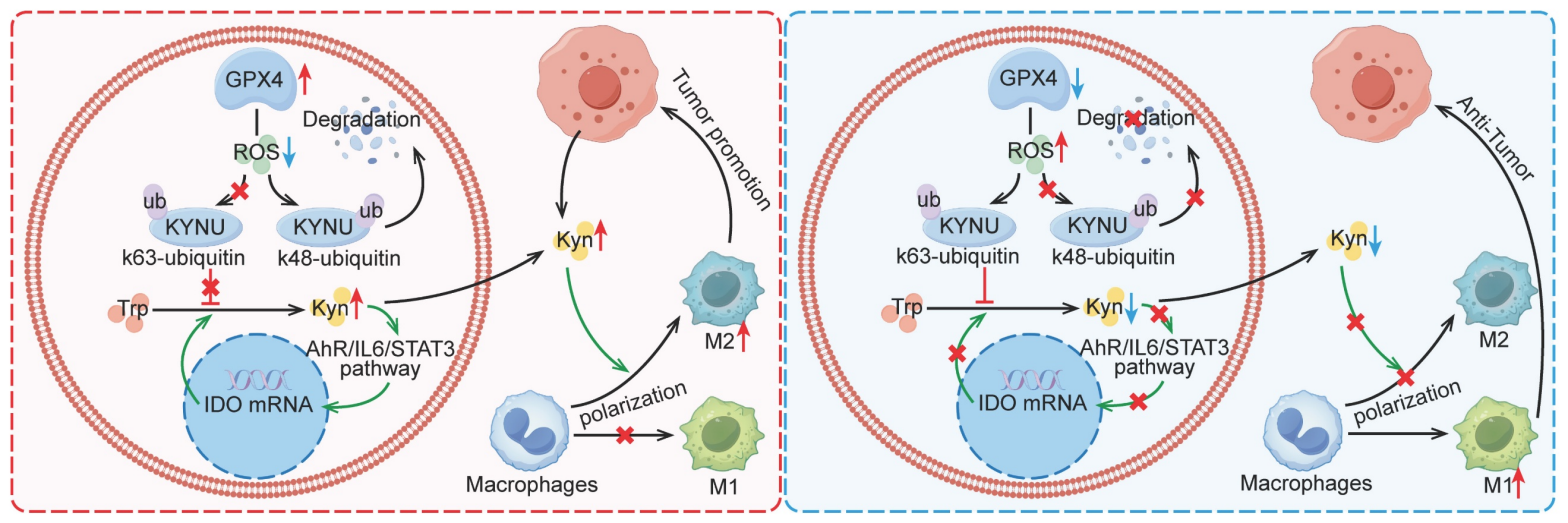
** Mechanism by which GPX4 in tumor cells regulates kynurenine metabolism via ROS, influencing macrophage polarization in the tumor microenvironment.** This graph illustrates the mechanism by which GPX4 knockdown in tumor cells affects kynurenine metabolism through the regulation of ROS levels, ultimately affecting macrophage polarization within the tumor microenvironment. Briefly, GPX4 knockdown in tumor cells increases intracellular ROS levels, which alters the ubiquitination pattern of KYNU, promoting its stabilization. This results in reduced intracellular and extracellular kynurenine levels. The decreased availability of kynurenine in the tumor microenvironment reduces M2 macrophage polarization and enhances M1 macrophage polarization, leading to an overall antitumor effect.

This study revealed that GPX4 in GC cells affected tumor growth via regulating tumor immune microenvironment, especially in tumor associated macrophage (TAM) polarization. Mechanistically, GPX4 knockdown elevates lipid peroxidation, triggering the conversion of KYNU ubiquitin chain modifications from K48 to K63. Such ubiquitination remodeling stabilizes KYNU expression (a key kynurenine-metabolizing enzyme), reduces kynurenine accumulation, and ultimately reprograms TAM polarization to enhance antitumor immunity. In addition, we also identified that the K96 and K163 sites are important for KYNU's modification by K48 and K63 ubiquitin chains **(Figure 8)**.

As a critical regulator of ferroptosis in tumors, GPX4 not only modulates tumor growth but also impacts tumor immune microenvironment. Recent evidence indicates inhibiting GPX4 expression in LAR subtype breast cancer induces ferroptosis in cancer cells and significantly increases the amounts of various T cell subtypes, including CD4^+^ and CD8^+^ T cells, switching the TME from "cold" to "hot" 35. Furthermore, GPX4 inhibition enhances the efficacy of ICIs 17. However, other studies have indicated that reduced GPX4 expression in colorectal adenocarcinoma (COAD) diminishes the efficacy of PD-1 blockade, which may be due to GPX4 activating cGAS-STING signaling, thereby inducing the infiltration of immune cells in COAD 18. The current study demonstrated that GPX4 in tumor cells significantly regulates the tumor immune microenvironment, inducing CD8^+^ T cell infiltration and modulating TAM polarization towards the tumor-promoting phenotype rather than the M1 phenotype in GC. We also found that macrophage depletion more strongly restored tumor size compared to CD8^+^ T cell depletion, suggesting that the pivotal role of macrophages in mediating the tumor-suppressive effects of GPX4 knockdown. As an important component of tumor microenvironment, TAM plays an important role in regulating T cell-mediated immune response. It is well known that M2 macrophages suppress CD8^+^ T cell proliferation and activity through the secretion of anti-inflammatory cytokines (e.g., CCL22 and TGF-β) 36,37 and the expression of immune checkpoint molecules (e.g., PD-L1) 38,39. Previous research has shown that both macrophages and kynurenine can inhibit CD8⁺ T cells, which implies that the increase in CD8⁺ T cells may be due to a reduction in M2 macrophage populations and/or decreased kynurenine levels following GPX4 knockdown. Additionally, GPX4 knockdown in tumor tissues was found to promote the secretion of pro-inflammatory factors (e.g., IL-1β, IL-6, IL-12, IFN-γ, and TNF-α) and inhibit the secretion of anti-inflammatory factors (e.g., IL-10 and TGF-β) in the blood, which is closely associated with tumor immune activation.

During tumor progression, TME cells and their secreted cytokines have pivotal functions in both supporting and inhibiting tumor growth. For instance, cancer cells produce considerable amounts of methionine transporters to deplete methionine in the TME, competitively inhibiting T cell access to methionine, thus affecting their survival and functions 40. Tumor-associated fibroblasts produce and secrete glutamine, which enters the microenvironment to enhance tumor cell energy metabolism and secrete glutaminase 1 microvesicles, promoting M2 macrophage polarization and inducing tamoxifen resistance 41. In this study, we observed a significant decrease in kynurenine levels following GPx4 knockdown in tumor cells via metabolomic analysis. Kynurenine plays a crucial role in cell growth 42. Intracellularly, tryptophan is metabolized into kynurenine through the rate-limiting enzymes indoleamine 2,3-dioxygenase 1 (IDO1), IDO2, and tryptophan 2,3-dioxygenase (TDO2). Kynurenine is in turn degraded by kynureninase (KYNU). In the TME, tumor cells "hijack" tryptophan, leading to a nutrient-deprived state for immune cells. Tryptophan-derived metabolites produced by IDO, particularly kynurenine, limit the proliferation and functions of multiple immune cells, e.g., T cells 43, NK cells 44, and macrophages 45, through various pathways including the aryl hydrocarbon receptor (AhR). In fact, M2 macrophages both produce and secrete significant amounts of kynurenine, contributing to immune suppression 46. Additionally, kynurenine produced by other cells in the TME can promote M2 macrophage polarization via the AhR/Nrf2 axis 47. Liu et al. 48 demonstrated that highly tumorigenic cancer stem cells deplete tryptophan in the TME via their high expression of IDO1, subsequently releasing metabolized kynurenine, which is taken up by T cells. This uptake promotes nuclear translocation of AhR in T cells, enhancing PD1 expression and impairing their immune-killing effects. The expression of IDO1 in cancer stem cells is stimulated by IFN-γ from T cells 48. Thus, targeting IDO1 in tumor cells with inhibitors could yield unexpected benefits 49. However, in phase III trials, the IDO1 inhibitor epacadostat did not enhance the therapeutic efficacy of the PD1 inhibitor pembrolizumab (Keytruda) 50. The current study shows that GPX4 in tumor cells regulates the ubiquitination and expression of the lesser-studied enzyme KYNU in the kynurenine pathway by controlling intracellular ROS levels, affecting kynurenine levels in the TME and ultimately affecting macrophage polarization. In addition, we also found that the reduction in kynurenine levels suppresses the AhR/IL6/STAT3 positive feedback pathway, thereby impairing kynurenine-producing enzyme IDO1 expression and further inhibiting kynurenine production.

Ubiquitination is a key post-translational modification that regulates both protein degradation and function, depending on the type of ubiquitin linkage. K48-linked ubiquitination is typically associated with protein degradation, as it marks substrates for recognition by the 26S proteasome, where they undergo degradation 51. The K48 chain is considered a signal for proteolytic processing, and it plays a central role in maintaining cellular homeostasis by regulating the turnover of damaged or misfolded proteins 52. In contrast, K63-linked ubiquitination does not lead to degradation but is primarily involved in regulating protein function. This type of ubiquitination often alters protein stability, activity, localization, or interaction with other molecules, and is crucial for processes such as signal transduction, DNA repair, and autophagy 53. For example, the K63-linked ubiquitination of ULK1, an autophagy-related kinase, enhances its stability and function, promoting the initiation of autophagy 33,34. Additionally, K63 chains are implicated in the regulation of many signaling proteins, such as IκBα and RIPK1, where they modulate signaling pathways without triggering protein degradation 54. These examples highlight the complex role of K63-linked ubiquitination in regulating protein function and stability in response to cellular stress or signaling events. In our study, we observed that ROS-induced GPX4 knockdown leads to a shift in KYNU ubiquitination from K48-linked chains to K63-linked chains. Specifically, during tumor cell growth, the K96 residue of KYNU is modified with K48-linked ubiquitin chains, which targets KYNU for proteasomal degradation. In contrast, under conditions of excessive ROS and oxidative stress, the K48-linked ubiquitination of KYNU is diminished, while the K163 residue is modified with K63-linked ubiquitin chains, ultimately reducing the degradation of KYNU. Moreover, our results suggest that the K63-linked ubiquitination of KYNU is closely associated with the stability of the protein. Previous studies have indicated that K48 and K63 ubiquitination on a protein might exist in a competitive relationship 55. This finding leads us to speculate whether there might be a competitive relationship between K48 and K63 ubiquitination on KYNU, as the K163 mutation in KYNU did not experience the stability increase typically caused by the inhibition of K48-linked ubiquitination by H_2_O_2_ treatment in the early time. However, it remains unclear whether the K96 and K163 sites of KYNU in this study are involved in crosstalk with other post-translational modifications, such as SUMOylation or NEDDylation, which can also influence protein localization, stability, and activity, as well as their participation in signaling pathway 56,57.

## Conclusion

In conclusion, this study demonstrates that GPX4 knockdown elevates lipid peroxidation, triggering a K48-to-K63 ubiquitination switch that stabilizes KYNU (a key kynurenine-catabolizing enzyme) through lysine residues K96 and K163, thereby reducing kynurenine accumulation and reprogramming TAM polarization toward an antitumor phenotype, which enhances CD8^+^ T cell-mediated immunity. Our study not only affirm the role of GPx4 in GC progression but also highlight it as a promising target for reshaping the immune microenvironment.

## Supplementary Material

Supplementary figures.

## Figures and Tables

**Table 1:** Primer sequences for RT-qPCR

Gene	Forward primer	Reverse primer
IDO1	AGTCGGAAGAGCCCTCAAAT	TGCCAGCCTCGTGTTTTATT
KYNU	GGAGGAATTGCTGGTGCCTT	TCTCTAAAGCTCTTGTCCTTGACT
GAPDH	CATGTTCGTCATGGGTGTGAA	CGCATGGACTGTGGTCATGAG
IL-10	TGTCCAGCTGGTCCTTTGTT	ACTGCACCCACTTCCCAGT
TGF-β	GAAACCCACAACGAAATCTATGAC	TTAACTTGAGCCTCAGCAGACG
TNF-α	CACAGTGAAGTGCTGGCAAC	AGGAAGGCCTAAGGTCCACT
VEGFA	GCACTGGACCCTGGCTTTACTGCTGTA	GAACTTGATCACTTCATGGGACTTCTGCTC
MMP9	GAAGGCAAACCCTGTGTT	AGAGTACTGCTTGCCCAGGA

**Table 2:** Spectral flow cytometry gating strategy

Cell type	Marker
Immune cells	CD45^+^
CD8^+^T cells	CD45^+^CD3^+^CD4^-^CD8^+^
CD8^+^T (PD1^+^) cells	CD45^+^CD3^+^CD4^-^CD8^+^PD1^+^
CD4+T cells	CD45^+^CD3^+^CD4^+^CD8^-^
Tregs	CD45^+^CD3^+^CD4^+^CD8^-^CD25^+^
B cells	CD3^-^B19^+^B220^+^
NK cells	CD3^-^CD19^-^B220^-^NK1.1^+^
DC cells	CD3^-^CD19^-^B220^-^NK1.1^-^CD11C^+^MHCII^+^
M1 type macrophage	CD3^-^CD19^-^B220^-^NK1.1^-^ CD11C^-^CD86^+^CD206^-^
M2 type macrophage	CD3^-^CD19^-^B220^-^NK1.1^-^ CD11C^-^CD86^-^CD206^+^
MDSCs	CD3^-^CD19^-^B220^-^NK1.1^-^ CD11C^-^CD86^-^CD206^-^Gr1^+^
