# Supplementary material for: GPX4 knockdown suppresses M2 macrophage polarization in gastric cancer by modulating kynurenine metabolism
Source: Theranostics. 2025 Apr 22;15(12):5826–45. doi: 10.7150/thno.108817 (PMC12068284; doi:10.7150/thno.108817)
Supplement: Supplementary file 1 — Supplementary figures. [file thnov15p5826s1.pdf]

Supplementary figures

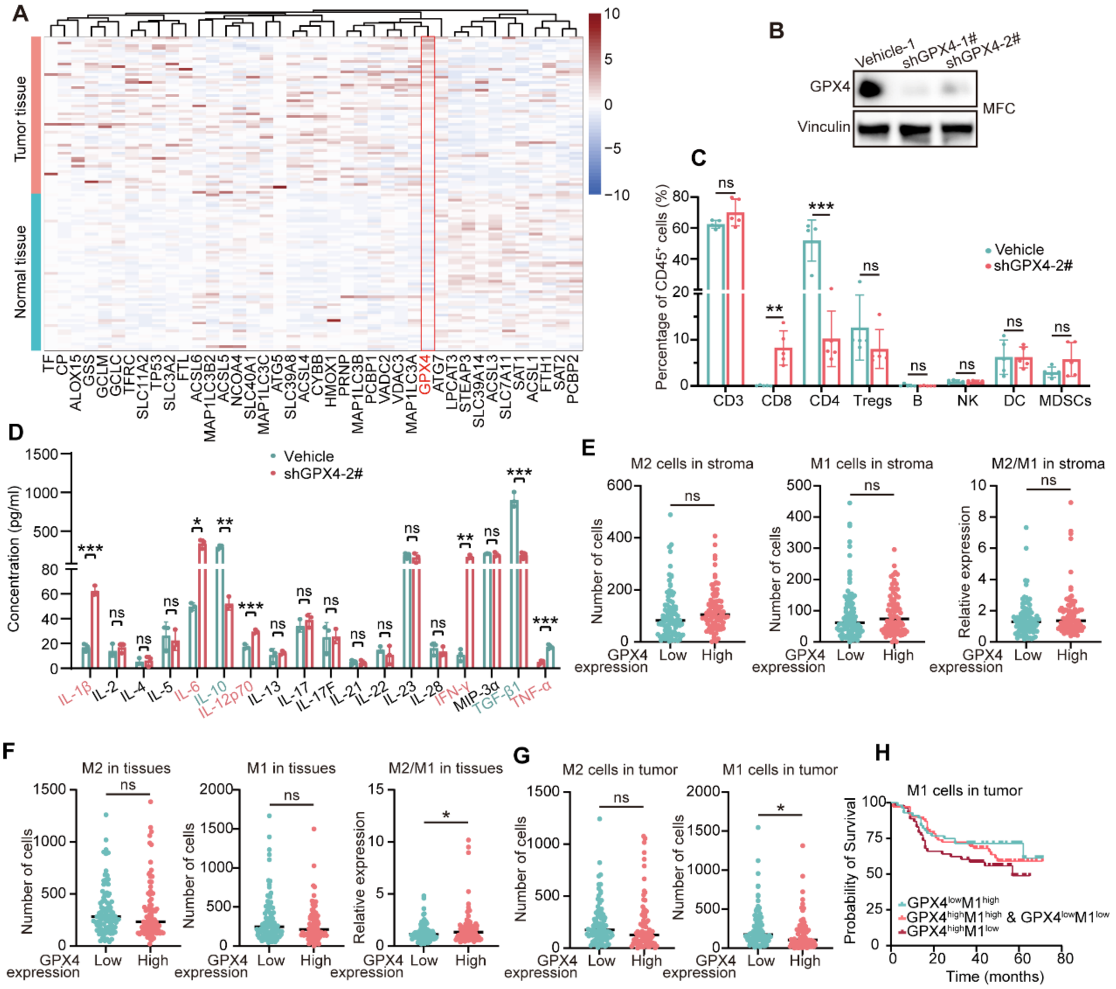

Figure S1. ***GPX4 Expression, Macrophage Infiltration, and Prognostic Outcomes in Gastric Cancer.*** **(A)** Heatmap of differentially expressed ferroptosis-related genes, including GPX4, in 60 pairs of GC and adjacent noncancerous tissue samples. **(B)** GPX4 knockdown efficiency in the murine GC cell line (MFC) assessed by western blot analysis. **(C)** Differences in immune cell populations, including CD8⁺ T cells and CD4⁺ T cells, between the GPX4-knockdown and vehicle groups, determined by spectral flow cytometry. **(D)** Expression differences of 18 cytokines in the peripheral blood of mice between the GPX4-knockdown and vehicle groups, detected by ELISA kit. **(E-F)** Associations of macrophage infiltration in the stromal compartment and entire tissue sections with tumor GPX4 expression levels, determined by tissue microarray analysis and multiplex immunohistochemistry. **(G)** Multiplex immunohistochemistry was performed to examine the relationship between GPX4 expression and M2 or M1 macrophage infiltration within the tumor tissue. **(H)** Prognostic analysis based on the associations of M1 macrophage levels with GPX4 expression in tumor tissues, assessing their combined impact on patient survival outcomes.

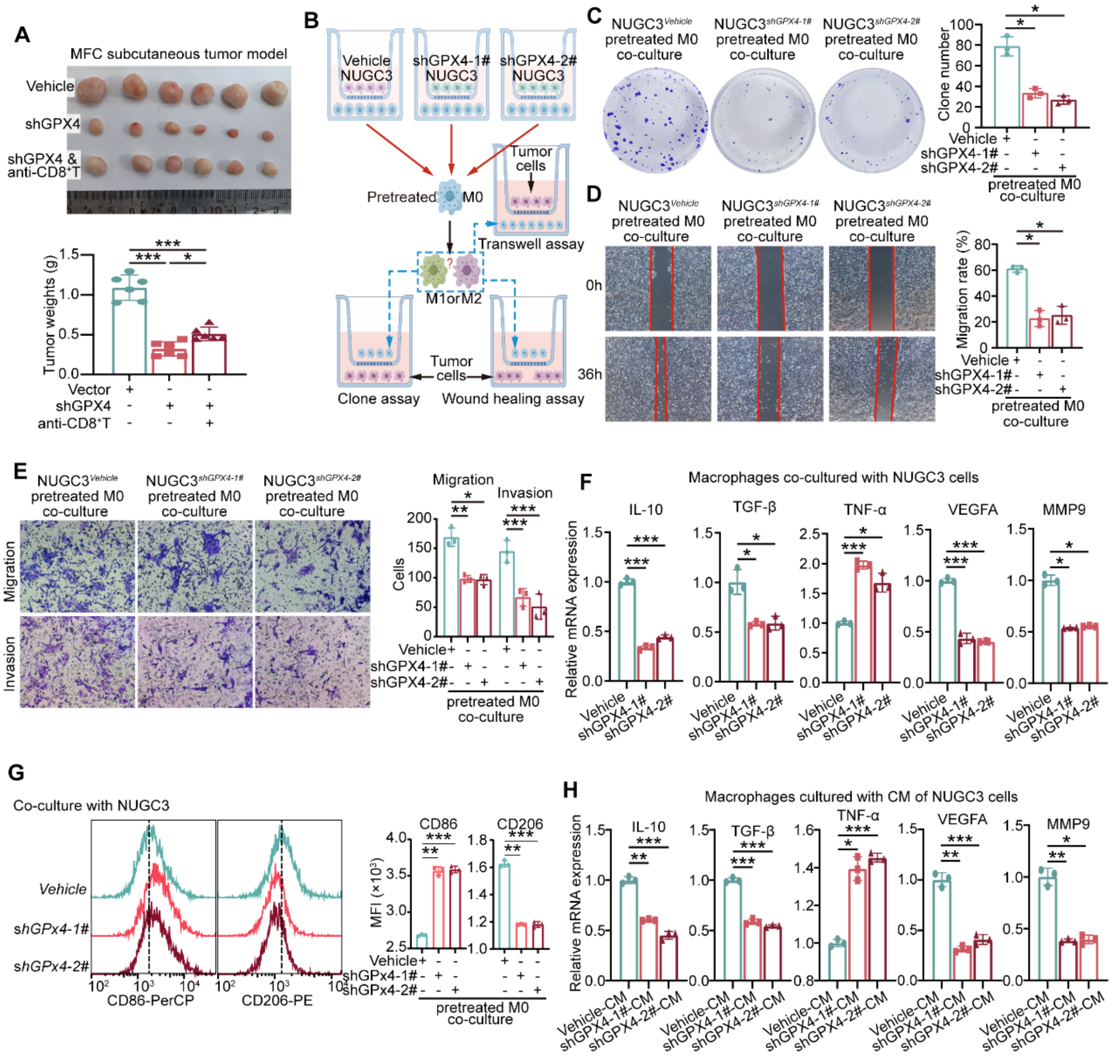

Figure S2. ***Impacts of GPX4 Knockdown on Macrophage Polarization and Tumor Cell Behavior in Gastric Cancer.*** **(A)** The MFC subcutaneous tumor model displays representative images and tumor weights from the vehicle group, GPX4 knockdown group, and GPX4 knockdown combined with anti-CD8^{+} T antibody treatment group. **(B)** Schematic diagram for investigating macrophage polarization changes and their functional impact. THP-1 cells, pretreated with 320 nM PMA for 6 h, were cultured with vehicle or GPX4-knockdown NUGC3 cells for 48 h. **(C-E)** Colony formation **(C)**, wound-healing **(D)**, and Transwell **(E)** assays in pretreated THP-1 cells co-cultured with GC cells. **(F–G)** RT-qPCR and flow cytometry analyses of IL-10, TGF-β, TNF-α, VEGFA, MMP9, as well as CD86 and CD206 expression in THP-1-derived macrophages after 48 h of co-culture with control NUGC3 cells and GPX4-knockdown NUGC3 cells. **(H)** RT-qPCR analysis of IL-10, TGF-β, TNF-α, VEGFA, and MMP9 expression levels in THP-1-derived macrophages after 48 h of culture with conditioned media from control NUGC3 cells and GPX4-knockdown NUGC3 cells.

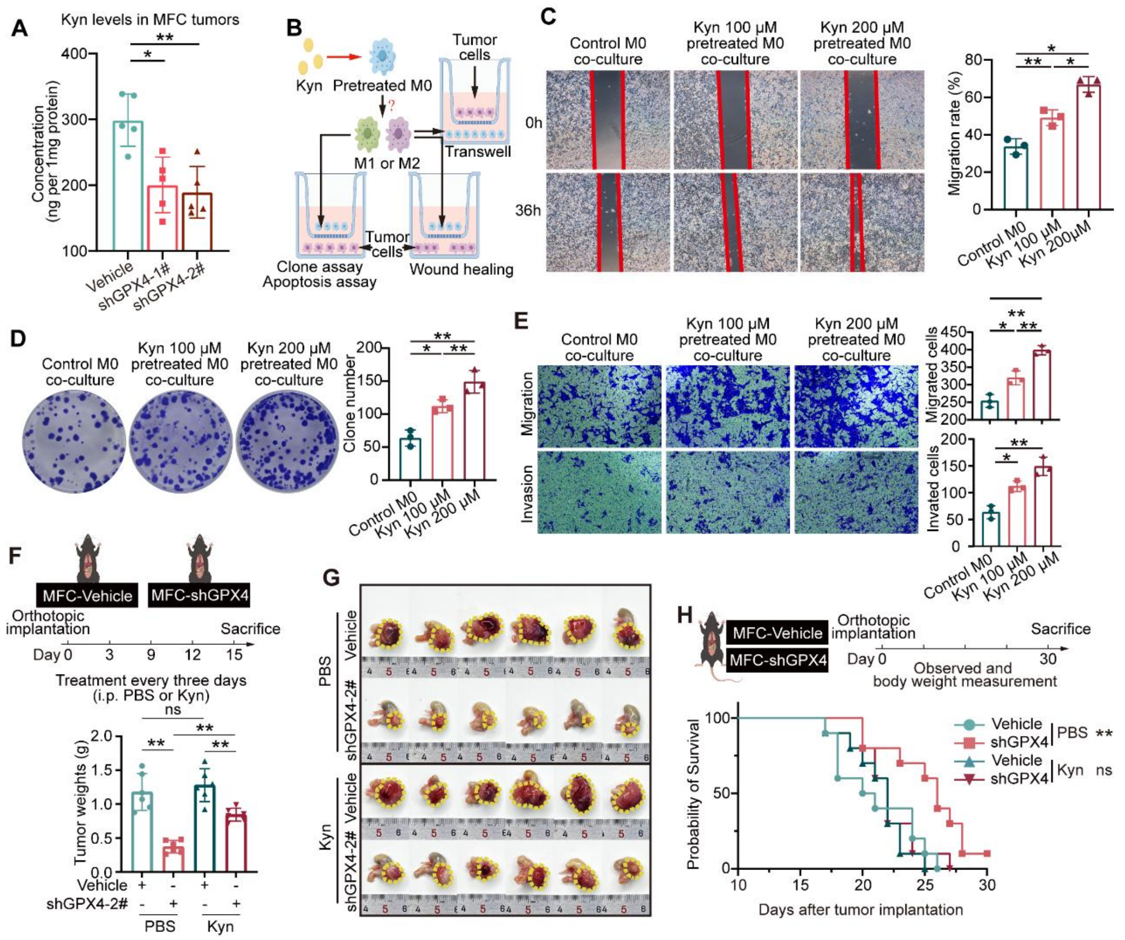

Figure S3. ***Effects of Kynurenine on Macrophage Function and Tumor Growth Dynamics.*** **(A)** Kynurenine levels in tumor tissues from GPX4-knockdown and vehicle MFC tumor-bearing mice. **(B)** Schematic diagram illustrating the investigation of macrophage polarization changes and their functional impact. THP-1 cells, pretreated with 320 nM PMA for 6 h, were exposed to varying concentrations of kynurenine for 48 h. **(C-E)** Wound-healing (**C**), colony formation **(D)**, and Transwell **(E)** assays in pretreated THP-1 cells subsequently co-cultured with NUGC3 cells. **(E)** Schematic representation of kynurenine treatment in mice to evaluate its role in GPX4 knockdown-induced tumor suppression. **(F-G)** Experimental setup showing the effects of GPX4 knockdown combined with kynurenine on tumor growth, as assessed by tumor weight and representative images, in an orthotopic GC mouse model. **(H)** Design of the orthotopic mouse model used to assess the survival benefits of GPX4 knockdown and the impact of kynurenine on survival outcomes.

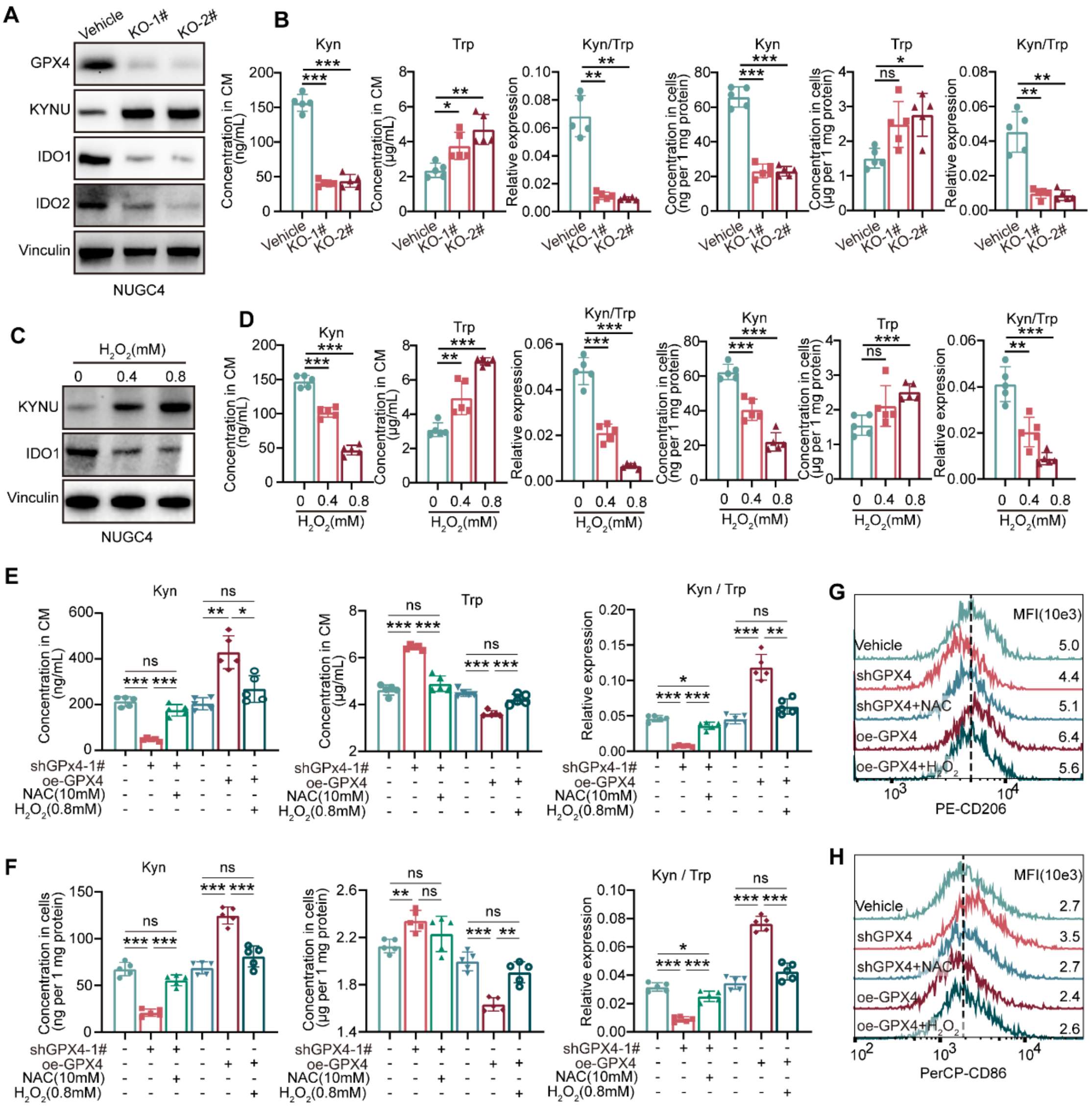

Figure S4. ***Role of ROS in GPX4-mediated kynurenine metabolism regulation and macrophage polarization.*** **(A-B)** GPX4 was knocked out in gastric cancer NUGC4 cells using sgRNA. Subsequently, the expression levels of KYNU, IDO1, and IDO2 proteins were measured, along with tryptophan, kynurenine, and the kynurenine/tryptophan ratio in both the culture supernatant and within the cells. **(C-D)** After treating NUGC4 cells with H_2O_2 for 6 h, the expressions of KYNU and IDO1, as well as the levels of tryptophan, kynurenine, and their ratio, were analyzed. **(E-F)**. After treating GPX4-knockdown NUGC3 cells with NAC (10nM) for 24 h and GPX4-overexpression NUGC3 cells with H_2O_2 (0.8mM) for 6 h, levels of kynurenine, tryptophan, and the kynurenine/tryptophan ratio were measured in conditioned medium and cells. **(G-H)** PMA-differentiated THP1 cells were treated with the above conditioned medium for 48 h, and flow cytometry was used to analyze CD206 and CD86 expressions.

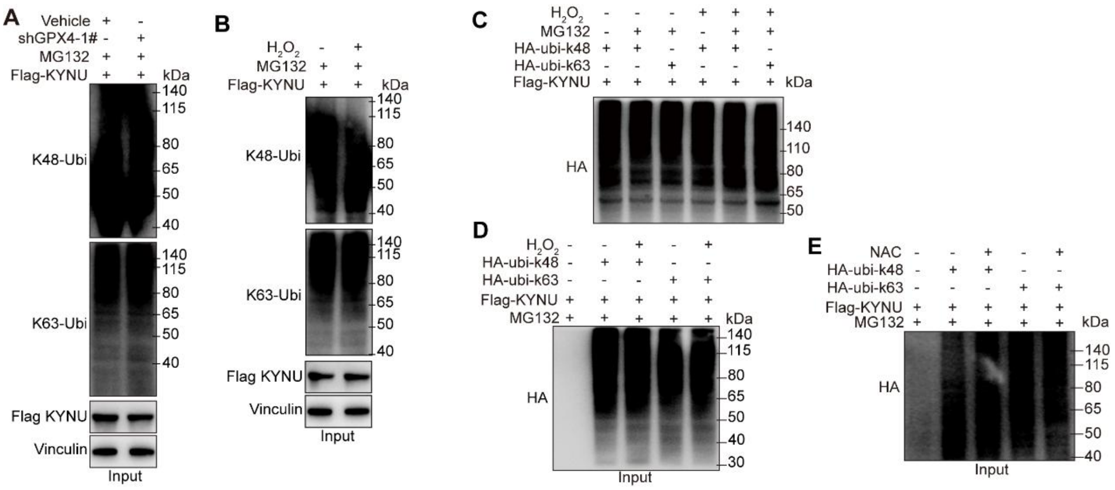

Figure S5. ***Co-IP Input Corresponding to the Experiments in Panels G (A), H (B), J (C), K (D), and L (E) of Figure 6.***
